# Plasma lipid profiling for the prognosis of 90-day mortality, in-hospital mortality, ICU admission, and severity in bacterial community-acquired pneumonia (CAP)

**DOI:** 10.1186/s13054-020-03147-3

**Published:** 2020-07-27

**Authors:** Mohammad M. Banoei, Hans J. Vogel, Aalim M. Weljie, Sachin Yende, Derek C. Angus, Brent W. Winston

**Affiliations:** 1 Department of Critical Care Medicine, Faculty of Medicine, Health Research Innovation Center (HRIC), University of Calgary, Room 4C64, 3280 Hospital Drive N.W, Calgary, Alberta T2N 4Z6 Canada; 2 Department of Biological Sciences, University of Calgary, Calgary, Alberta Canada; 3 Department of Pharmacology, University of Pennsylvania, Philadelphia, PA USA; 4 The Clinical Research, Investigation, and Systems Modeling of Acute Illness (CRISMA) Laboratory, University of Pittsburgh, Pittsburgh, PA USA; 5 Department of Critical Care Medicine, University of Pittsburgh, Pittsburgh, PA USA; 6 Departments of Medicine and Biochemistry and Molecular Biology, Health Research Innovation Center (HRIC), University of Calgary, Room 4C64, 3280 Hospital Drive N.W., Calgary, Alberta T2N 4Z6 Canada

**Keywords:** Plasma metabolomics, Lipid profiling, Bacterial CAP pneumonia, Mortality prediction

## Abstract

**Introduction:**

Pneumonia is the most common cause of mortality from infectious diseases, the second leading cause of nosocomial infection, and the leading cause of mortality among hospitalized adults. To improve clinical management, metabolomics has been increasingly applied to find specific metabolic biopatterns (profiling) for the diagnosis and prognosis of various infectious diseases, including pneumonia.

**Methods:**

One hundred fifty bacterial community-acquired pneumonia (CAP) patients whose plasma samples were drawn within the first 24 h of hospital admission were enrolled in this study and separated into two age- and sex-matched cohorts: non-survivors (died ≤ 90 days) and survivors (survived > 90 days). Three analytical tools, ^1^H-NMR spectroscopy, GC-MS, and targeted DI-MS/MS, were used to prognosticate non-survivors from survivors by means of metabolic profiles.

**Results:**

We show that quantitative lipid profiling using DI-MS/MS can predict the 90-day mortality and in-hospital mortality among patients with bacterial CAP compared to ^1^H-NMR- and GC-MS-based metabolomics. This study showed that the decreased lysophosphatidylcholines and increased acylcarnitines are significantly associated with increased mortality in bacterial CAP. Additionally, we found that decreased lysophosphatidylcholines and phosphatidylcholines (> 36 carbons) and increased acylcarnitines may be used to predict the prognosis of in-hospital mortality for bacterial CAP as well as the need for ICU admission and severity of bacterial CAP.

**Discussion:**

This study demonstrates that lipid-based plasma metabolites can be used for the prognosis of 90-day mortality among patients with bacterial CAP. Moreover, lipid profiling can be utilized to identify patients with bacterial CAP who are at the highest risk of dying in hospital and who need ICU admission as well as the severity assessment of CAP.

## Introduction

Community-acquired pneumonia (CAP) is of global importance to medicine with less than 5% mortality in outpatients and 5–10% and 25–30% mortality in hospitalized patients and intensive care unit (ICU) patients, respectively. Mortality can reach 50% among those who are in septic shock and require vasopressors [1, 2]. CAP was reported as the seventh most common cause of mortality in the USA with 50,000 deaths in 1.5 million hospitalized patients each year [3–5]. The morbidity of CAP is between 0.3 and 0.5% worldwide in adults [6]. Management of CAP patients is often complicated due to poor quality evidence of clinical data such as radiographic findings, difficulty with accurate diagnosis, poor prognostic signs, and non-specific therapeutic strategies that make the prediction of patient outcomes uncertain [7]. There is a global interest in predicting short-term (< 90 days) and long-term (1 year) CAP mortality [2]. Current severity and mortality assessment methods such as scoring systems and biomarkers are not sensitive or specific enough to predict mortality accurately. APACHE II/III, SAPS, SOFA, and PSI are commonly used scoring systems for the prognosis and severity assessment of CAP that can enhance the prediction of prognosis in association with other current biomarkers such as procalcitonin (PCT) and C-reactive protein (CRP) [1]. Several scoring systems, such as PSI, APACHE II, CURB-65, and SAPS [8], have been used to categorize severity and predict short-term and long-term mortality of CAP in association with two putative biomarkers: PCT and CRP [9]. Metabolomics, both non-targeted and targeted approaches, provide a powerful tool to identify and quantify low molecular weight compounds (metabolites) in biofluid samples that contribute to normal and pathological pathways as primary, intermediate, and/or end products of metabolism [10, 11]. Metabolites, and their biopatterns, are being used as biomarkers for the diagnosis, prognosis, and prediction of mortality in critically ill patients [12, 13]. In this retrospective observational matched cohort study, we aimed to examine the prediction or prognosis of 90-day mortality among patients with bacterial CAP and identify those who are at the highest risk of dying in hospital (in-hospital mortality) using a multi-platform metabolomic approach. We applied non-targeted proton nuclear magnetic resonance (^1^H-NMR) spectroscopy, gas chromatography mass spectrometry (GC-MS), and targeted direct infusion tandem mass spectrometry (DI-MS/MS) to analyze metabolomic biopatterns of plasma samples for CAP patients collected within 24 h of admission to hospital for prognostication of mortality.

## Materials and methods

### Study subjects

One thousand eight hundred ninety-five patients were enrolled in the community-acquired Genetic and Inflammatory Markers of Sepsis (GenIMS) study, a retrospective and multicenter study (28 sites) in southwestern Pennsylvania, Connecticut, southern Michigan, and western Tennessee (the cohort was originally published by Kellum et al. in 2007) [14]. In total, 150 CAP patients from the original cohort were enrolled in this study based on positive bacterial cultures and/or plasma concentration of PCT > 0.25 ηg/ml (strongly suggestive of bacterial infection) during the first 24 h of admission to hospital and radiographic evidence of pneumonia. Of these, 75 were non-survivors who died ≤ 90 days after admission and 75 were age-, sex-, and PCT level-matched (as per GenIMS) individuals who survived CAP infection > 90 days. More information about the enrolled subjects and patient’s characteristics is available in Table 1. Non-survivors that included 26 patients who died before hospital discharge were defined as in-hospital deaths. Table 1 also displays clinical features and patients’ characteristics of the in-hospital death cohort compared to survivors. Also, 31 ICU-ventilated controls without CAP were enrolled for comparison in the diagnostic part of the study. These were individuals admitted to the ICU that did not have pneumonia. They were predominantly post-op cardiovascular surgery or neurosurgery patients admitted for routine ventilation post-op prior to extubation.

**Table 1:** Clinical characteristics of 150 bacterial CAP patients (non-survivors *n* = 75 vs. survivors *n* = 75) and in-hospital death cohort (*n* = 26)

Variables	Survivors (***n*** = 75)	Non-survivors (***n*** = 75)	In-hospital death (***n*** = 26)
Age years (mean ± SD)	78.6 ± 8.8	78 ± 8.7	76.7 ± 10.5
Male/female	31/44	31/44	7/19
Weight (mean ± SD)	158 ± 36.8	147.5 ± 42.3	145.63 ± 50.4
Hospital LOS	7.17 ± 4.1	9.85 ± 7.4	11.1 ± 11.4^Ŧ^
ICU LOS	0.76 ± 2.3	1.97 ± 3.3	3.9 ± 4^Ŧ^
APACHE III	60.4 ± 15.2	73.7 ± 20.6*	82.9 ± 24.4^Ŧ^
PSI (day 0)	77.2 ± 5.7	97.8 ± 51.7*	121 ± 46.9
PSI (day 1)	112.4 ± 31.4	134.5 ± 38.7*	157.8 ± 41.7^Ŧ^
PSI (day 1 no age)	38.2 ± 26.9	60 ± 38*	84.0 ± 41.0^Ŧ^
Mechanical ventilation^a^	4 (5.3)	20 (26.6)*	15 (57.6)^Ŧ^
Noninvasive ventilation^a^	5 (6.6)	12 (16)*	3 (11.5)
Comorbidities^a^			
Other respiratory diseases	25 (35)	33 (44)	9 (34)
Neoplastic diseases	4 (5)	7 (9)	3 (11)
Neurological diseases	10 (7)	17 (22)*	2 (8)
Aids	0 (0)	1 (1.3)	1 (4)
Sepsis	19 (32)	30 (47)	14 (53)^Ŧ^
Liver disease	0 (0)	1 (1.6)	1 (4)
CHF	19 (33)	15 (24)	6 (25)
Cerebrovascular disease	10 (5)	10 (6)*	1 (4)
Renal disease	3 (5.7)	7 (11)	3 (12)^Ŧ^
Altered mental status	6 (10.5)	11 (18)*	8 (33)^Ŧ^
Smoker	50 (66)	51 (68)	19 (73)
Alcoholism	22 (29)	16 (21)	5 (19)
Pregnancy	21 (16)	10 (13)	1 (4)
Clinical manifestation^a^			
Lowest temperature (°C)	36.43 ± 0.58	36.46 ± 2.1	36.39 ± 2.5
Highest temperature (°C)	37.20 ± 0.68	37.27 ± 0.88	37.15 ± 0.81
Pulse ≥ 125/min	6 (8)	12 (16)	6 (23)
BUN ≥ 30 mg/dl	12 (16)	27 (36)*	15 (57)^Ŧ^
Respiratory rate/min	9 (15)	17 (27)	9 (34)^Ŧ^
PO^2^ < 60 mm/Hg	21 (36)	24 (44)	13 (50)
pH < 7.35	3 (4)	4 (6)*	4 (15)
Lowest systolic BP (mm/Hg)	118 ± 19	116 ± 24	115 ± 21
Highest systolic BP (mm/Hg)	146 ± 22	148 ± 25	149 ± 23
Highest creatinine (mg/dl)	1.27 ± 0.79	1.92 ± 1.59	1.95 ± 1.52

*SD* standard deviation, *LOS* length of stay, *APACHE III* Acute Physiology and Chronic Health Evaluation used as an ICU scoring system, *PSI* Pneumonia Severity Index, *CHF* Congestive Heart Failure. ^a^Data is no. (%) of subjects, unless otherwise indicated; *significant difference between non-survivors and survivors; ^Ŧ^significant difference between in-hospital death cases and survivors

### Study design

This retrospective, case-control study was designed using two cohorts enrolled at the Universities of Calgary and Pittsburgh. The plasma-based metabolomics of non-survivor’s (≤ 90 days) and survivor’s (> 90 days) cohorts were compared to each other to examine whether metabolomics is associated with the prognosis of mortality of bacterial CAP. To identify the patients who are at the highest risk of dying inside the hospital using metabolomics, 26 patients who died in-hospital were compared to 75 patients who survived (> 90 days) with bacterial CAP. To determine whether metabolomics can be used for diagnosis of bacterial CAP, 40 CAP patients admitted to the ICU were compared to 30 no CAP ICU controls.

### Metabolomic profiling

Three analytical platforms, ^1^H-NMR, GC-MS, and DI-MS/MS, were applied to identify metabolites for 150 bacterial CAP patient plasma samples. Since a single analytical technique is less able to identify and quantify a broad range of metabolites from different chemical classes, we used three common analytical techniques to cover a larger number of metabolites. Non-targeted one-dimensional ^1^H-NMR spectroscopy was performed using a 600-MHz Bruker Ultrashield Plus NMR spectrometer (Bruker BioSpin Ltd., Canada) as previously described [13]. Fifty-five metabolites including sugars, sugar alcohols, amino acids, and organic acids were quantified using ^1^H-NMR spectroscopy. The ^1^H-NMR spectra were profiled using ChenomX NMR Suite 7.1 software (ChenomX Inc., Edmonton, Alberta, Canada) [15]. We used an Agilent chromatograph 7890A (Agilent Technologies, USA) coupled with a Waters GCT mass spectrometer to acquire GC-TOF-MS spectra. GC-MS was performed as previously described [13]. Compound detection was performed using metabolite detector software [16]. One hundred eighty-five features were detected by the GC-MS platform which included 85 structurally known and 100 unknown compounds including sugar alcohols, alpha-keto acids, and organic acids. Quantitative DI-MS/MS was performed using an ABI 4000 Q-Trap tandem mass spectrometry instrument (Applied Biosystems/MDS Analytical Technologies, Foster City, CA) equipped with a solvent delivery system. The Absolute IDQTM p150 kit (BIOCRATES Life Sciences AG, Innsbruck, Austria) was used for the targeted quantification consisting of 150 known metabolites, including 88 glycerol-phospholipids [phosphatidylcholines (PCs), aa=diacyl, ae=acyl-alkyl, lysophosphatidylcholines (lysoPCs)], 33 acylcarnitines [AC, (Cx: y)], 15 sphingolipids (SM x: y), 14 amino acids, and one sugar. The *MetIQ*™ software package (BIOCRATES Life Sciences AG, Innsbruck, Austria) was applied to quantify metabolites as described by the manufacturer.

### Data analysis

Principal component analysis (PCA) was performed to observe intrinsic differences between samples and potential cohort aggregation, trends, similarities, and outliers among the two cohorts. Supervised orthogonal partial least squares discriminant analysis (OPLS-DA) was applied to predict mortality by discrimination of two cohorts based on the most differentiating metabolites. The OPLS-DA models were verified by three performance parameters *R*^2^*Y*, *Q*^2^*Y*, and *p* value which are obtained by cross-validation (CV) method. These indicators are used for assessing reliability and measuring the significance level of a model. CV is based on the leave-one-out cross-validation (LOOCV) that is used to validate the models. It is also known as internal cross-validation or internal validation. *R*^2^*Y* and *Q*^2^*Y* indicate goodness of fit and goodness of prediction, respectively. Although the *Q*^2^ > 0 is considered to have predictive relevance for the prediction model [17], a value of ≥ 0.3 was defined as having prediction value for the models based on the human samples [18]. A permutation test was applied for the validation and prevention of overfitting of the OPLS-DA models based on 200 random permutations. For the OPLS-DA models, variable important in projection (VIP) analysis was performed to select the most differentiating metabolites/features for the separation of the two groups in a weighted fashion. Coefficient plots were also created to observe metabolites/features and their relative correlations between the two cohorts with metabolites exhibiting increased or decreased concentration in each group. Partial least square regression (PLSR) was applied as a linear multivariate approach to finding the relationship of the most differentiating metabolites and prediction of mortality. Scoring systems such as APACHE III and PSI were also used for prediction of mortality [19]. As part of the LOOCV internal validation, a misclassification analysis on the predicting group (randomly selected ¼ of samples) was carried out to measure sensitivity, specificity, and AUROC using SIMCA-P v15.0.2.

Univariate statistical analyses were applied as a complementary statistical analysis to enhance the amount of information and maximize the extraction of relevant information from the metabolomic study datasets. Univariate strategies provide a direct measure of significance, the *p* value and false discovery rate (FDR), and non-parametric tests that are useful in metabolomics [20]. The *T* test and Wilkinson-Whitney test were used to assess the significance of the separation between non-survivors and survivors based on variables with or without normal distribution, respectively.

## Results

### Patient characteristics

Table 1 shows the demographics, clinical information, and comorbidities of the two CAP groups (75 non-survivors vs. 75 survivors). The mean (± SD) age was 78.6 (± 8.8) and 78.6 (± 8.8) for the non-survivor and survivor groups, respectively. Thirty-one males and 44 females were included in both the survivor and non-survivor cohorts (Table 1). As expected, there were differences in hospital and ICU lengths of stay (LOS), APACHE III, and PSI scores in day 0 (day of admission) and day 1 between non-survivors and survivors. Table 1 also shows the demographic and clinical characteristics of the in-hospital death patients vs. survivors. There are statistically significant differences between the in-hospital deaths and survivors for hospital LOS and ICU LOS, APACHE III score, and PSI scores for day 1 and mechanical ventilation. Moreover, we observed statistically significant differences between the two groups in the presence of the following comorbidities: sepsis, renal diseases, and altered mental status. Using samples on the first day, 40 patients had a positive culture with known bacterial causes, equally distributed among non-survivors (*n* = 20) and survivors (*n* = 20). The bacterial causes consisted of 13 different species *Streptococcus pneumonia*, *Staphylococcus aureus*, *Escherichia coli*, *Pseudomonas aeruginosa*, *Acinetobacter baunmannii*, *Klebsiella pneumonia*, *Moraxella catarrhalis*, *Serratia marcescens*, *Haemophilus influenzae*, unspecified *Haemophilus*, *Enterobacter cloacae*, *Enterobacter aerogenes*, and *Listeria monocytogenes*.

### Analysis of metabolite patterns showed that lipid profiling can predict mortality among patients with bacterial CAP better than other metabolites

Statistical analysis showed metabolite profiles obtained by quantitative DI-MS/MS were more informative for the prognosis of mortality among patients with bacterial CAP when compared to the ^1^H-NMR and GC-MS metabolomic platforms (data not shown). The DI-MS/MS metabolite dataset was substantially more predictive, significant, sensitive, and specific.

Table S10 summarizes several prediction model parameters of the three analytical platforms used in this study, showing the advantage of DI-MS/MS and the significance of lipid metabolites. Moreover, a good agreement was found between univariate and multivariate data analysis on the DI-MS/MS dataset by extracting a more specific and consistent pattern for lipid alterations to separate non-survivors from survivors that are discussed here.

Based on the DI-MS/MS data compared to the ^1^H-NMR and the GC-MS data, the role of lipid profiling obtained by DI-MS/MS on prognosis of bacterial CAP will be primarily presented in this manuscript.

### Can we prognosticate 90-day mortality of bacterial CAP using lipid profiling?

**Fig. 1:**
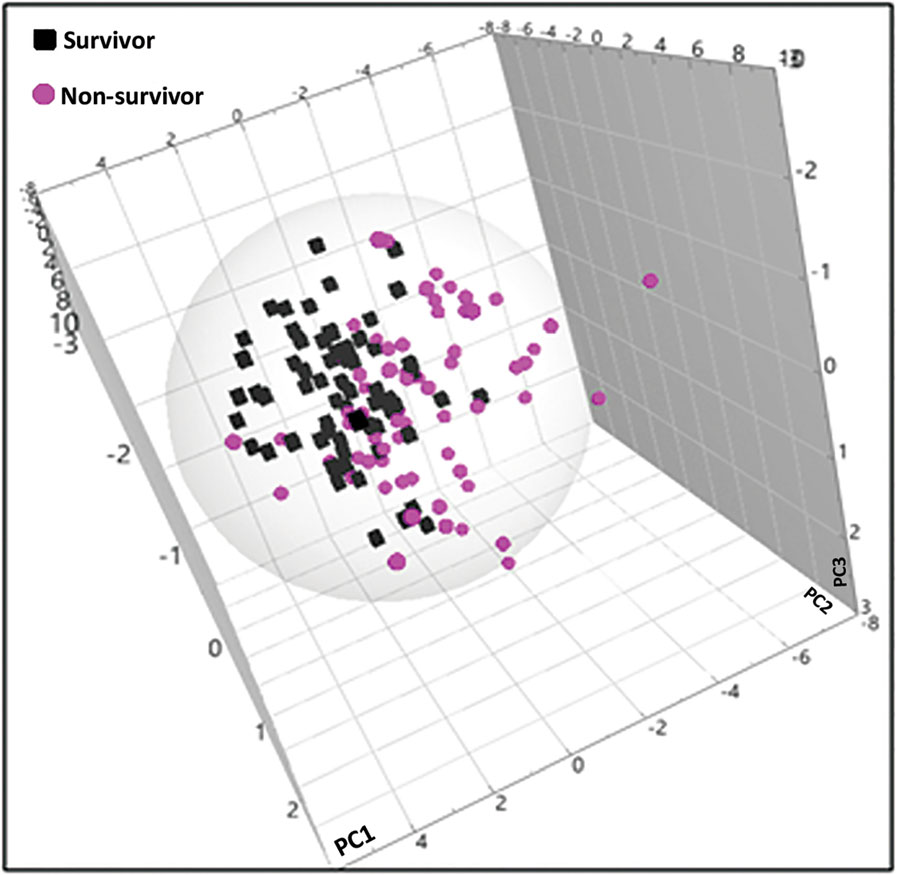
PCA analysis of DI-MS/MS day 1 plasma metabolites comparing non-survivors (*n* = 75) to survivors (*n* = 75) of bacterial CAP patients. The cumulative *R*^2^*X* = 0.554 showed a high variability between two cohorts

PCA analysis showed a clustering between non-survivors (≤ 90 days) and survivors (> 90 days) with an *R*^2^*X* = 0.554 based on the lipid profiling (Fig. 1). The differences amounted to 95% of the total variation in the dataset, indicating that the metabolomic characteristics of the two groups were different.

**Fig. 2:**
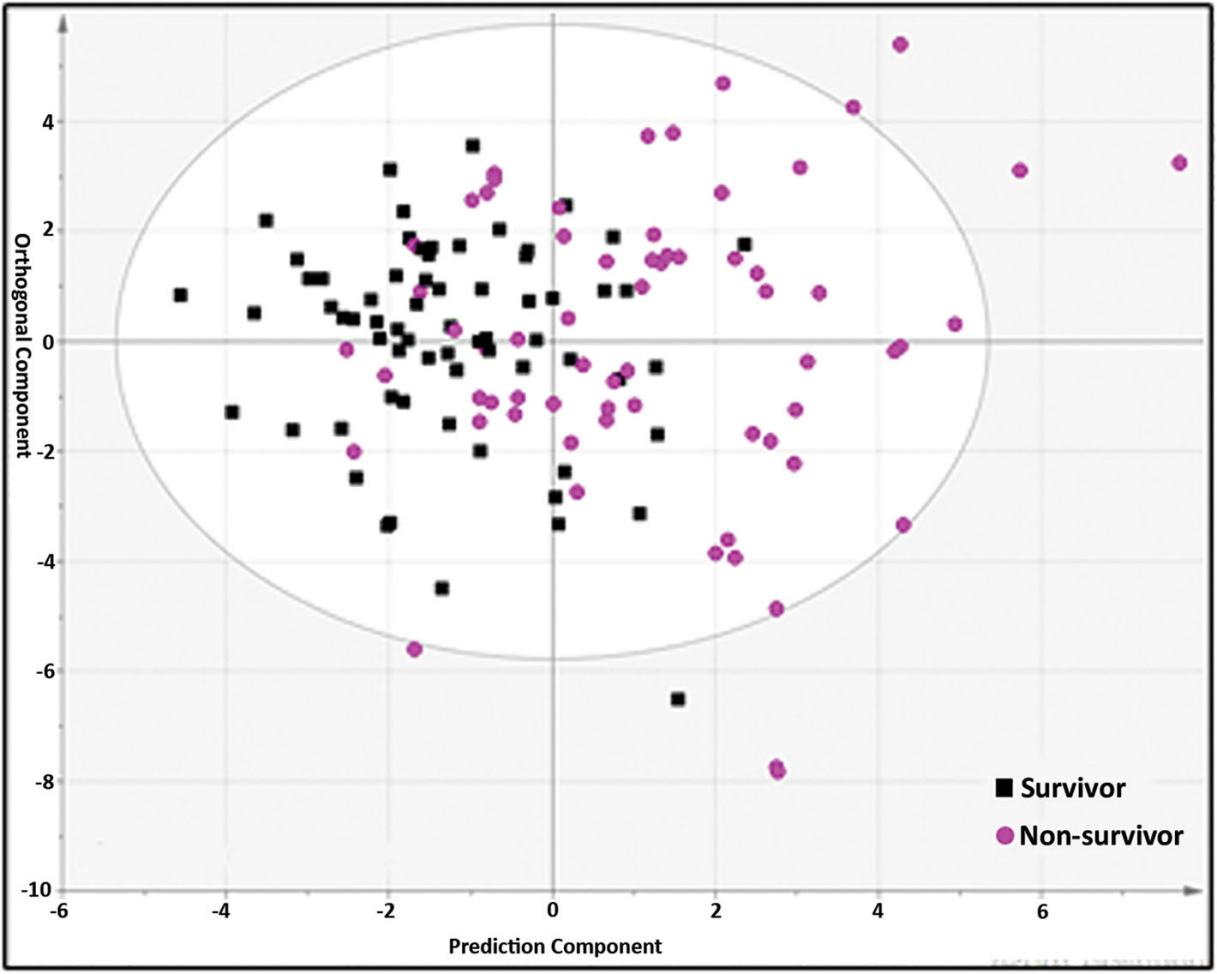
OPLS-DA analysis of DI-MS/MS day 1 plasma metabolites comparing non-survivors (*n* = 75) to survivors (*n* = 75) of bacterial CAP patients. This shows a predictable model with a high statistical significance using VIP > 1.0 including 20 metabolites (*R*^2^*Y* = 0.331, *Q*^2^*Y* = 0.299, *p* = 1.15 × 10^−8^)

**Fig. 3:**
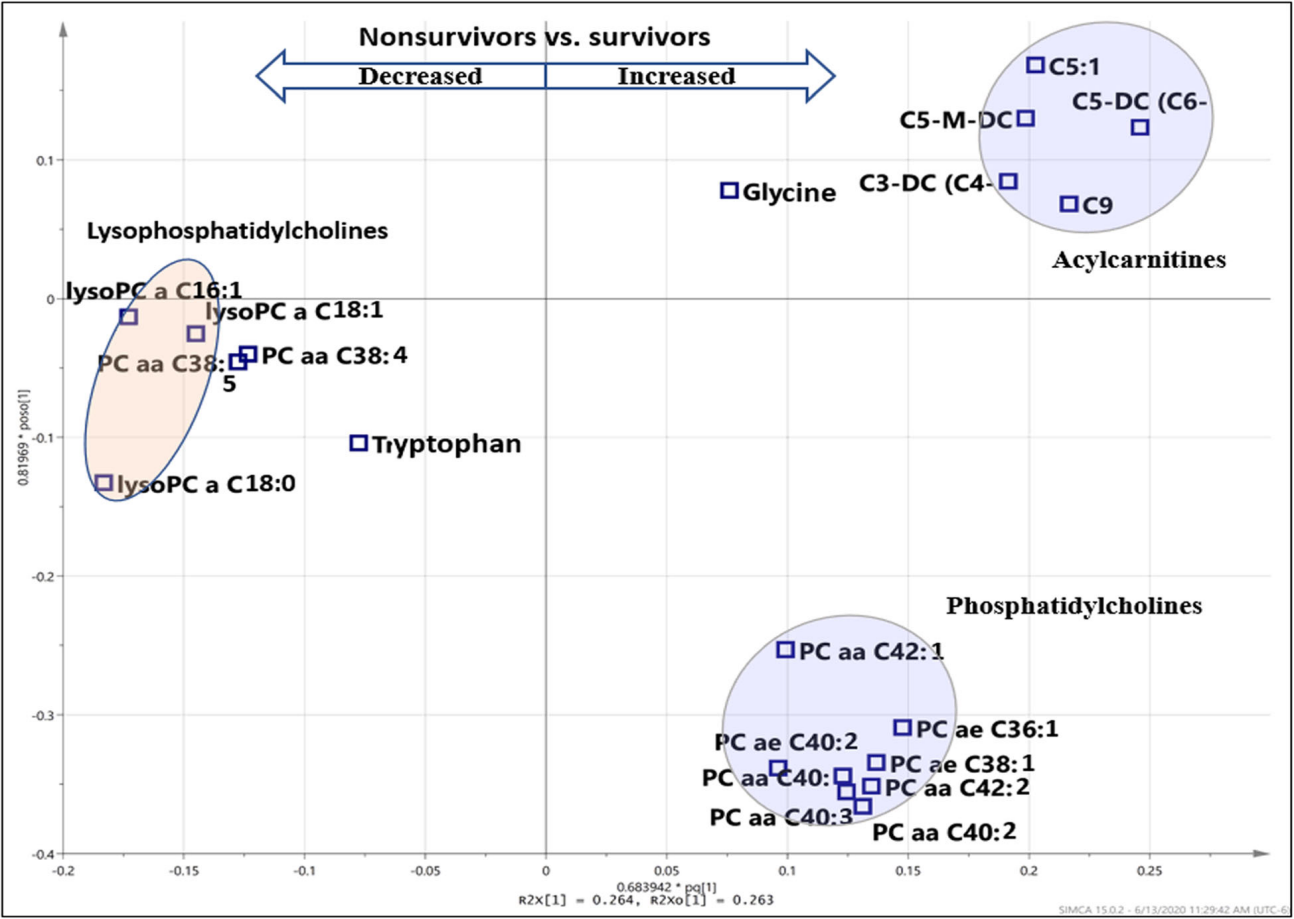
Loading plot shows that increased acylcarnitine and decreased lysophosphatidylcholine compounds in non-survivors (≤ 90-day mortality) compare to survivors (> 90-day mortality)

The mortality among bacterial CAP patients was predicted by separation of non-survivors (*n* = 75) from survivors (*n* = 75) using the most differentiating metabolites (VIP > 1.0) in an OPLS-DA analysis (Fig. 2). The *Q*^2^*Y* = 0.299 value for lipid dataset shows a predictive capability of metabolites to separate 90-day non-survivors from survivors. This shows a mortality prognostic value of metabolomics based on plasma samples drawn on the 1st day of admission to the hospital. In total, 20 metabolites contributed to the prognosis of 90-day mortality in bacterial CAP (Table 2). This statistical model to separate two cohorts had 82% sensitivity, 91% specificity, and AUROC of 0.91. The relative concentration of metabolites and their changes between survivors and non-survivors have been illustrated by the coefficient plot (Fig. S1). It has been observed that AC, lysoPC, and PC compounds are strongly correlated to each other and contribute in the separation of the two cohorts (Fig. 3).

**Table 2:** DI-MS/MS based on 20 important metabolites (VIP > 1.0) that contributed to separate 90-day non-survivors from survivors

Quantified metabolites by DI-MS/MS		
	Increased in non-survivors	Decreased in non-survivors
1	C5-DC (C6-OH) (glutaryl-l-carnitine)	Tryptophan
2	C3-DC (C4-OH) (malonyl carnitine)	LysoPC a C18:0
3	C5-M-DC (methylglutaryl-l-carnitine)	LysoPC a C18:1
4	C5:1 (tigyl-l-carnitine)	PC aa C38:5
5	Glycine	PC aa C38:4
6	C9 (lysophophatidylethanolamine, nonayl-l-carnitine)	LysoPC a C16:1
7	PC ae C40:2 (glycerol 3-phosphocholine)	
8	PC aa C42:1 (lecithin, PC)	
9	PC aa C40:3	
10	PC ae C36:1	
11	PC ae C38:1 (lecithin, phosphatidylcholine)	
12	PC aa C40:1 (lecithin, PC)	
13	PC aa C42:2 (lecithin, PC)	
14	PC aa C40:2	

This shows the increased and decreased metabolites in non-survivors vs. survivors. The order of metabolites reflects the relative amount of change

**Fig. 4:**
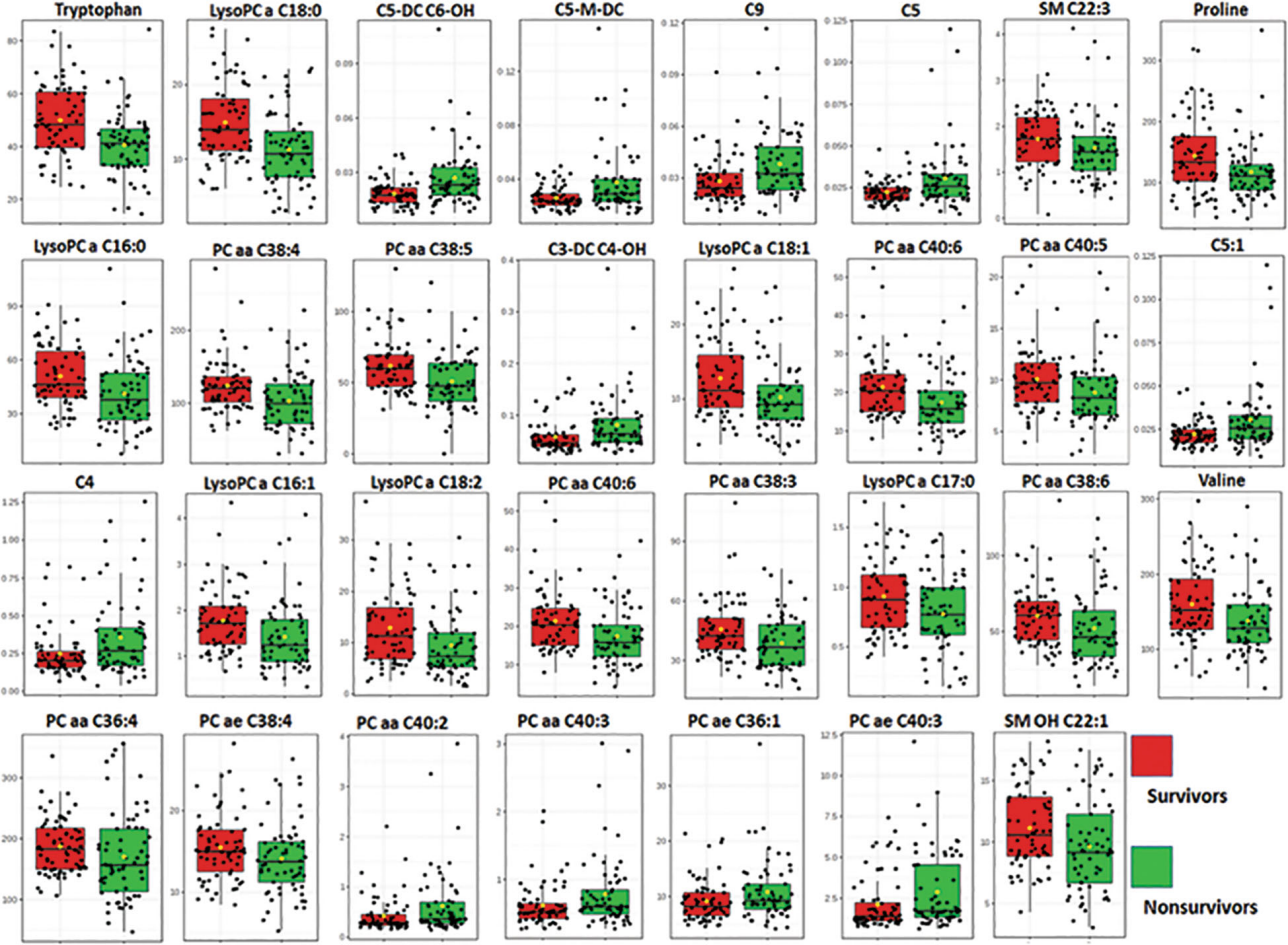
Metabolite concentration plot comparing non-survivor vs. survivor of bacterial CAP. The unpaired *t* test shows 31 metabolites with significant changes (FDR < 0.05) between 90-day non-survivors and survivors using DI-MS/MS. Table S2 shows all metabolites with significant *p* value (< 0.05)

Similar to MVA, a UVA approach using an unpaired *t* test on the most differentiating metabolites demonstrated 32 metabolites that were significantly (FDR *q* < 0.05) different between non-survivors and survivors (Fig. 4 and Table S1).

Although the *Q*^2^ to predict mortality using an MVA approach was not too high (0.299), the prediction model possessed a highly significance *p* value and was validated with the use of permutation test (200 times) (Fig. S2), strongly suggesting the data are not overfit. Moreover, these findings become more predictive when unpaired *t* test analysis is performed, a completely different analytic approach than MVA, showing similar trends in the changes of the most important metabolites; this is discussed in the following section. PLS-regression also showed a very strong relation (*R*^2^ = 0.95) between the most differentiating metabolites (*n* = 20) obtained by the prediction model and separation of non-survivors from survivors (Fig. S3). This strong relation can prove the value of lipid profiling to predict mortality using plasma samples of the 1st day of admission.

### Do lysophosphatidylcholines (lysoPCs) and acylcarnitines (ACs) act as biomarkers for prognosis of 90-day mortality of CAP pneumonia?

Metabolic profile using multi- and univariate analyses, two different approaches, showed a decrease of all measured lysoPCs in non-survivors. Using multivariate analysis, we observed a decrease in 3 lysoPCs, C18:0, C18:1, and C16:1, out of the 20 most differentiating metabolites in the prediction model (Table 2). The *T* test analysis (Table S1) also shows 8 lysoPCs including lysoPC C16:0, C16:1, C17:0, C18:0, C18:1, C18:2, C20:3, and C20:4 which were significantly (FDR < 0.05) reduced in non-survivors. Moreover, all AC compounds involved in the discrimination were significantly increased in non-survivors compared to survivors. We observed an increased level of the most differentiating AC compounds (*n* = 5) including C5-DC, C3-DC, C5-M-DC, C5:1, and C9, observed by multivariate analysis and by univariate analysis with a significant increase (FDR < 0.05) in ACs (including C3-DC (C4-OH), C5-DC (C6-OH), C5-M-DC, C5:1, C10:2, C8, C2, C7-DC, C4, and C8:1). Multivariate loading plot and heatmap analysis (Fig. 3) show that AC and PC compounds are inter-correlated.

These findings are validated by the fact that the most differentiating metabolites obtained by multi- and univariate analysis, two different approaches, change in the same direction between non-survivors and survivors except for PC ae C40:0 that changed in the opposite direction between the survivor and non-survivor cohorts.

### Can we predict in-hospital mortality among patients with bacterial CAP using lipid profiling?

We further investigated the use of metabolomics in patients with bacterial CAP to determine who are at the highest risk of dying in the hospital. Plasma-based lipid profiling of patients who died before hospital discharge (in-hospital mortality) was compared to survivors (> 90 days).

**Fig. 5:**
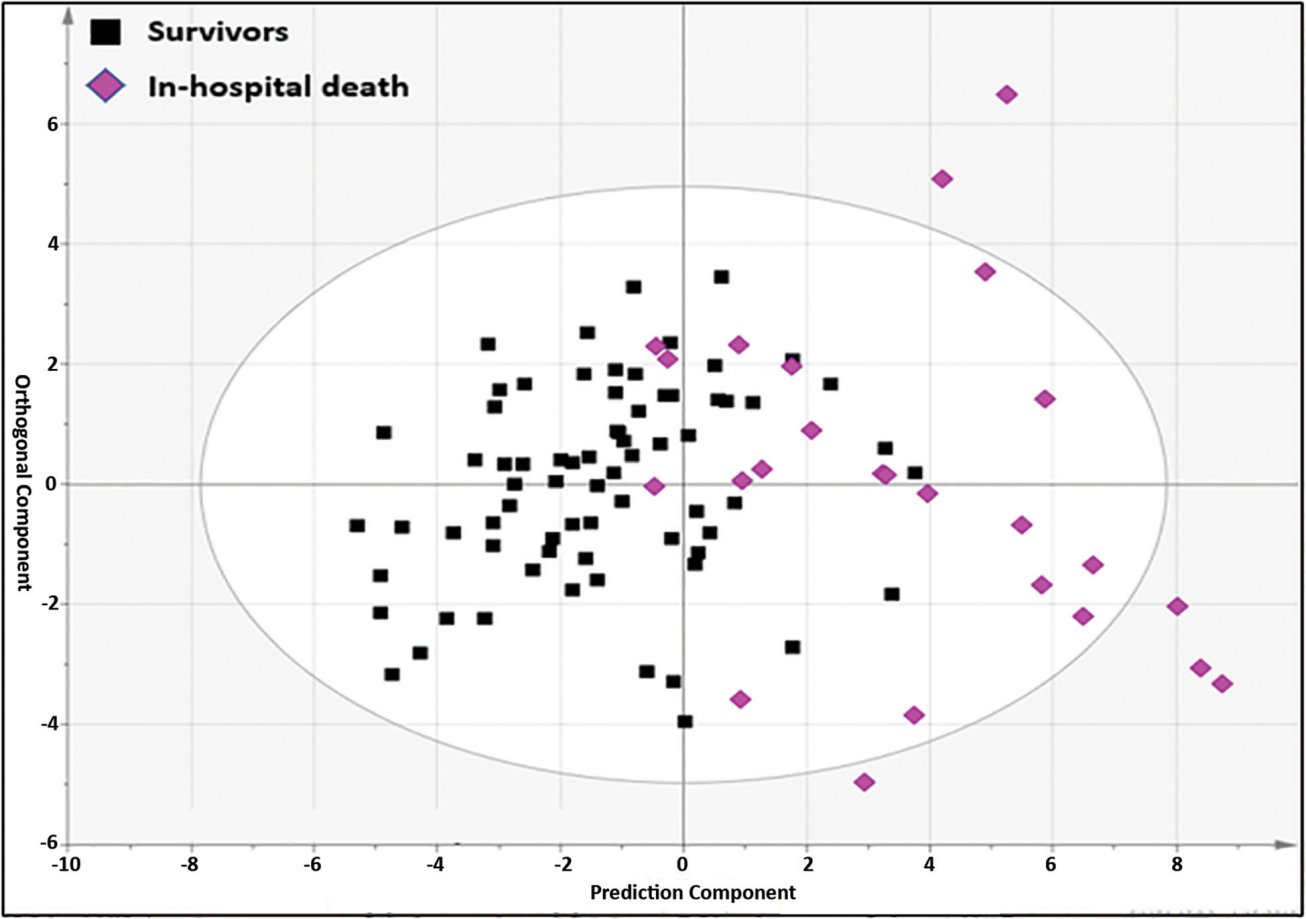
DI-MS/MS-based OPLS-DA model to separate in-hospital deaths from survivors using 22 metabolites with VIP > 1.0. *R*^2^*Y* = 0.501, *Q*^2^*Y* = 0.433, *p* = 9.91 × 10^−11^

**Fig. 6:**
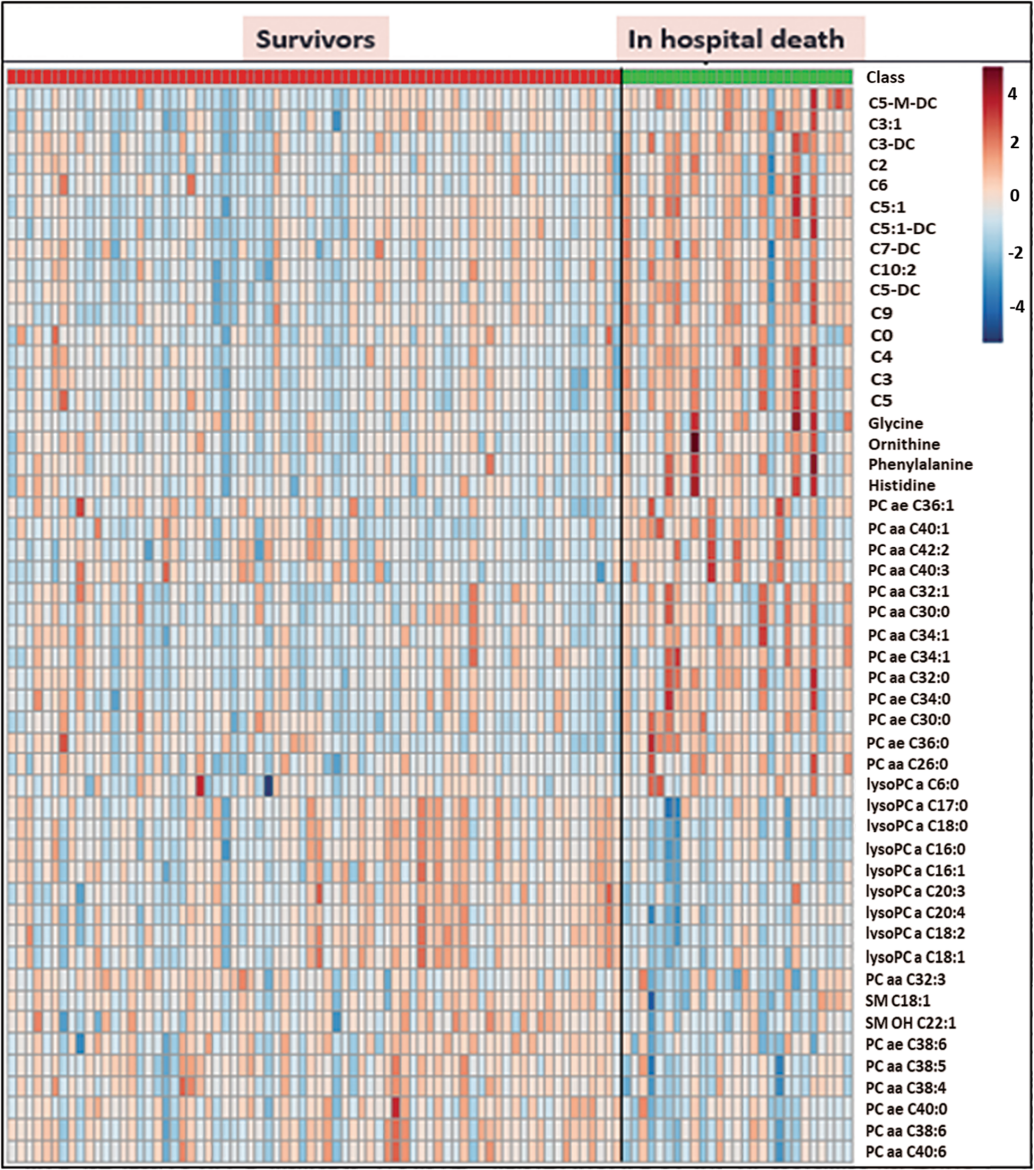
Heatmap analysis shows a separation between in-hospital deaths and survivors using the most differentiating metabolites by DI-MS/MS

Table 1 shows the information on 26 patients who died before hospital discharge (in-hospital mortality) as a subgroup of non-survivors. The metabolomic results showed that in-hospital mortality can be predicted using metabolite alterations of patients who died in-hospital (*n* = 26) compared to survivors (> 90 days, *n* = 75). The in-hospital death cohort was differentiated from the survivor cohort with “relatively good predictability” (*Q*^2^*Y* = 0.433) based on the lipid profiles obtained by the DI-MS/MS platform (Fig. 5). Twenty-one lipids and 1 amino acid (phenylalanine) contributed to the separation of two cohorts. This discrimination model was highly sensitive (96%) and specific (90%) with a high AUROC of 0.96. The coefficient plot provided the relative concentration of selected metabolites in the discrimination of both cohorts (Fig. S4). The unpaired *t* test analysis revealed that both cohorts are significantly different in metabolic profile with 65 metabolites having an FDR < 0.05 comparing in-hospital deaths and survivors (Table S2). A heatmap clearly illustrated the difference between the two cohorts based on the most differentiating lipids and fatty acids (Fig. 6). A very strong correlation (*R*^2^ = 0.94) was found between the most differentiating metabolites and separation of in-hospital deaths from survivors using PLS-regression analysis (Fig. S6).

### Can lysophosphatidylcholines, phosphatidylcholines, and acylcarnitines be used as biomarkers for predicting in-hospital mortality of bacterial CAP?

**Fig. 7:**
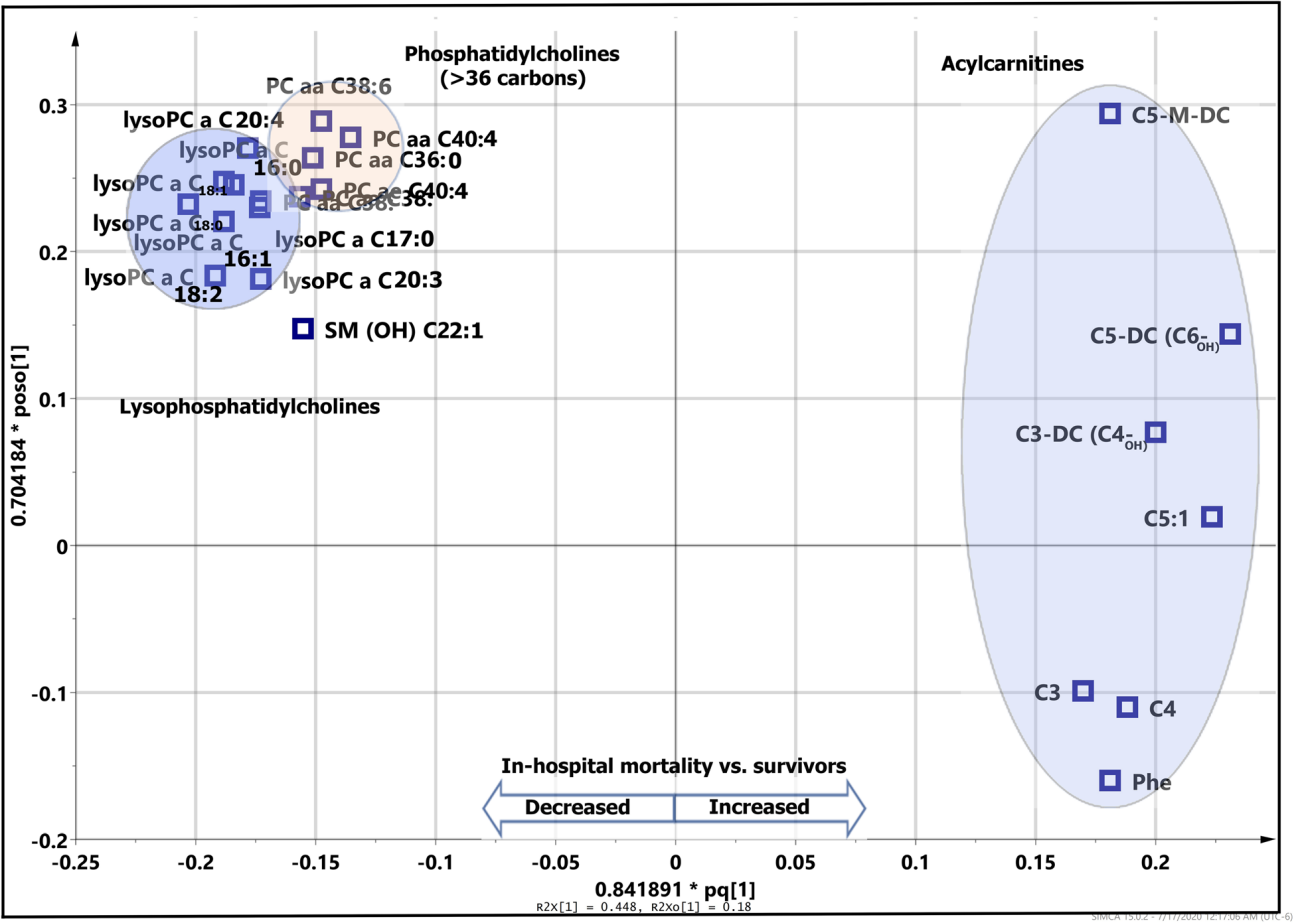
Loading plot shows correlation of metabolites belonging to different lipid classes and how the metabolites can be used to separate in-hospital death from survivors. Acylcarnitines increase while phosphatidylcholine (> 36 carbons) and lysophosphatidylcholines decrease in the in-hospital mortality cohort compared to the survivor cohort

Both MVA and UVA analyses demonstrated a consistent pattern of a significant decrease of lysoPCs in patients who died in-hospital (in-hospital mortality) when compared to survivors (Fig. 7). Coefficient plot (Fig. S4) and *t* test analysis (Table S2) all show lysoPCs are differentiating compounds including lysoPC C16:0, C16:1, C17:0, C18:0, 18:1, C18:2, C20:3, and C20:4 that all decrease in the in-hospital mortality cohort compared to survivors. The comparison also revealed increased short-chain AC compounds (C3, C3-DC, C4, C5, C5:1, C5:1-DC, C5-M, and C9) as well as decreased medium- and long-chain ACs (C14:1, C14:2, C16 C18, and C18:1) in the in-hospital death cohort vs. survivors.

The univariate *t* test analysis showed that the most differentiating PC compounds were markedly decreased in the in-hospital death cohort versus survivor (Table S2). Nonetheless, a multivariate approach revealed PC compounds with more than 36 carbons were significantly decreased in the in-hospital mortality cohort vs. survivor cohort. PCaaC38:0, PCaeC38:6, PCaa40:6, PCaa38:6, PCae40:0, PCae38:0, PCaaC38:4, PCaeC38:4, and PCaaC38 were decreased in the in-hospital death cohort.

Figure 7 also shows the correlation and integration of metabolites belonging to each fatty acid class in the prediction of in-hospital mortality. Although in-hospital deaths showed higher APACHE III score, hospital LOS, ICU LOS, and PSI scores compared to survivors, it was demonstrated that lysoPCs, PCs, and ACs could predict in-hospital mortality on the 1st day of hospital admission through plasma-based metabolomics using the DI-MS/MS platform.

In fact, current data show the high potency of quantitative lipid profiling for the prognosis of mortality in CAP whether with or without clinical features.

### Strong relationships were found between lipid profiling and phenotypic traits: APACHE III and PSI

Multivariate regression (PLS regression) analysis revealed a strong correlation (*R*^2^ > 0.9) between the most differentiating lipid metabolites and two phenotypic traits APACHE III at day 0 and PSI at days 0 and 1 (Fig. S8 A-D). Although APACHE III and PSI are significantly different between non-survivors and survivors, logistic regression analysis showed they have a lower correlation with separation of non-survivors and survivors, lacking predictability compared to lipid metabolites (Table S3).

### Can we predict the need for ICU admission using lysophosphatidylcholines and acylcarnitines?

Our data showed that 40 out of 150 patients with bacterial CAP were admitted to ICU with the length of stay from 1 to 14 days. This data also revealed that 36% (*n* = 27/75) and 18.7% (*n* = 14/75) of non-survivors and survivors were admitted to the ICU, respectively. ICU admission was 69.2% among in-hospital deaths. Lipid profiling was analyzed among 40 patients who were admitted to ICU vs. 40 age- and sex-matched patients without ICU admission. Table S11 shows the analysis of patients’ characteristics that required admission to the ICU versus those who did not need ICU admission. As it was expected, the ICU LOS, mechanical ventilation, and noninvasive ventilation frequencies among non-ICU-admitted cohorts were zero, thus different than non-ICU patients. The only other significantly different variable was hospital LOS between ICU- and non-ICU-admitted cohorts.

The results demonstrated that 22 metabolites significantly (*p* < 0.05) changed between ICU and non-ICU patients (Table S6). Interestingly, we observed decreased lysoPCs and increased AC compounds in ICU patients compared to non-ICU patients. This result highlights a potential role of lysoPCs and ACs as biomarkers to distinguish patients who need ICU admission for the bacterial CAP. Remarkably, these compounds showed a similar significant change to prognosticate 90-day and in-hospital mortality.

### Are acylcarnitines, lysophosphatidylcholines, and phosphatidylcholines associated with pneumonia severity?

We further investigated the association of lipid profiles with pneumonia severity using the PSI score on the day of admission. Based on the five risk classes of PSI score, the bacterial CAP cohort was divided into less severe (PSI grades 1–3) and more severe (PSI grades 4–5) groups. Results demonstrated that 37 metabolites were significantly changed between the two groups including 12 metabolites with a FDR < 0.05 (Table S7). This analysis showed increased ACs and decreased lysoPCs and PCs among patients with more severe bacterial pneumonia compared to patients with less severe disease. This difference has clearly been reflected in heatmap analysis (Fig. S11). This analysis revealed that ACs and lysoPCs can be used as predictor biomarkers for determining the severity of CAP.

### Diagnosis of ICU-admitted CAP pneumonia from ICU-ventilated control cohort

To demonstrate the value of plasma-based metabolomics for the diagnosis of CAP pneumonia from ICU-ventilated control, 40 out of 150 bacterial CAP patients with ICU admission history were compared to 30 ICU-ventilated controls. Table S4 lists the patient’s characteristics and hospital and ICU LOS. Both ^1^H-NMR and GC-MS data were applied to metabolomically differentiate ICU-admitted bacterial CAP from ICU-ventilated control. The results showed a very distinctive metabolic signature between the two cohorts. OPLS-DA analyses revealed 55 metabolites and 114 features obtained by ^1^H-NMR and GC-MS, respectively, that contributed to the separation of the two cohorts (Fig. S9 and S10). The predictability of discrimination of bacterial CAP from ICU control was high for NMR (*Q*^2^*Y* = 0.777) and GC-MS (*Q*^2^*Y* = 0.852) methods with very good statistically significant differences between two cohorts with high sensitivity, specificity, and AUROC (Table S5). The *T* test analysis revealed 27 metabolites and 46 known features significantly changed (FDR *q* < 0.05) between bacterial CAP when compared to ICU-ventilated controls using ^1^H-NMR and GC-MS, respectively (Table S8 and S9).

## Discussion

The present study was designed to examine the role that metabolomics might play in the prognosis of mortality among patients with bacterial CAP. In a comprehensive analysis, the results of this study showed that lipid compounds offer important insights into the prognosis of 90-day mortality of bacterial CAP on the 1st day of admission to the hospital. Moreover, we also demonstrated that lipid profiling is capable to predict in-hospital mortality from survivors (> 90 days) using a plasma sample drawn on the 1st day of admission to the hospital. Our result provides important insight into the prediction of mortality in patients who are at the highest risk of dying in hospital. In a targeted approach using DI-MS/MS, lysoPCs and ACs were prognostic metabolites for the mortality of bacterial CAP when compared to survivors. Correspondingly, decreased lysoPCs, increased ACs, and decreased PCs significantly changed yielding a fingerprint to prognosticate in-hospital mortality. Besides the prognosis of mortality of bacterial CAP, we also showed that targeted lipid profiling using a DI-MS/MS platform could be used to predict the need for ICU admission and assessment of severity for the patients with bacterial CAP. Interestingly, decreased lysoPCs, increased ACs, and decreased PCs were associated with severity and ICU admission requirement.

Power analysis using multivariate data analysis (using R-based analysis) [21] showed that using the most differentiating metabolites, 24 samples in each group could provide a significant difference between cohorts (FDR < 0.05) with a power *β* = 0.8 for the study. Thus, the enrollment of 75 samples in each group indicates the study should have sufficient power to detect a difference between groups with 80% power.

To our knowledge, this is the first study to identify lipids and related metabolites as potential predictors of in-hospital mortality in CAP patients as well as prediction of ICU admission and assessing CAP severity. Metabolite biomarkers can be used independently or to supplement other approaches like genomics and proteomics biomarkers or even clinical scoring systems in precision medicine. Lipids are very diverse biomolecules comprising a range of different classes including ACs, glycerophospholipids, SMs, sterol lipids, and glycerolipids. Lipids can play important roles in many biological and physiological functions such as structural components of cell membranes, intermediates in signaling pathways, homeostasis, and immunity [22]. Once these studies are externally validated, these findings have a promising capability of being translated into clinical practice. Primarily, DI-MS/MS can be considered as a shotgun technique to quantify limited number or hundreds of metabolites per sample in a very short period of time (hours). Using the quantitative and targeted approaches shown in this study will allow one to develop a new prognostic tool for bacterial CAP investigations and prognostication. In addition, this study showed that the semi-quantitative and untargeted GC-MS and NMR analytical platforms appear to be not efficient enough at this time to be prognostically helpful for bacterial CAP. Importantly, this study showed that lipids are important metabolites for the prognosis of 90-day mortality and in-hospital mortality while other metabolites quantified in this study such organic acids, amino acids, amines, sugars, and sugar alcohols were not as predictive of mortality in bacterial CAP. Specifically, lipid compounds including saturated and unsaturated fatty acids are in close relationship with mechanisms of inflammation, the central essential mechanisms of the host response to bacterial infections such as seen in bacterial CAP [23]. They are active substances and important inflammatory mediators in both pro-inflammatory and anti-inflammatory mechanisms [24, 25]. On the other hand, 90% of surfactant in the lung is formed by lipids and PCs make up more than 80% of these lipids. Pneumonia may cause surfactant changes leading to an alteration in lipid metabolism [26]. The low concentration of lysoPCs in non-survivors may be caused by the consumption of lysoPCs in the early stages of disease or by conversion of lysoPCs by phospholipase A due to increased secretion of autotoxins [27]. PCs are also major components of the lipid bilayers of cell membranes as well as lung surfactant and both are important in lung development [28]. Alveolar type II cells are responsible for the synthesis and accumulation of PCs in the lung [29]. It is assumed that damage to the cell membrane, alveolar cell integrity, and surfactant dysfunction due to inflammatory illness such as pneumonia is associated with increased PCs in the blood that could be correlated with the severity and mortality of CAP. Indeed, several studies have shown changes of lipid concentration in the blood after acute lung injury due to sepsis and bacterial and viral infections [30, 31]. All of the former strongly suggests that lipids may be interesting targets as putative biomarkers for the prognosis of mortality of CAP in clinical practice.

The current findings are consistent with those reports showing the importance of fatty acids and lipids in the diagnosis and prognosis of CAP and other respiratory complications such as ARDS and septic shock. For example, low- and high-density lipoprotein cholesterol (LDL-C and HDL-C) were found to be independent predictors for bacterial CAP adverse outcomes [22]. In addition, the alterations of some fatty acids such as docosahexaenoic acid, eicosapentaenoic acid, and oleic acid were associated with increased and decreased risk of CAP in women and men [32, 33].

Several studies suggest that an increase of AC compounds occurs in patients with CAP. Overall, previous studies have shown increased short-, medium-, and long-chain ACs in CAP patients compared to non-CAP patients [34] and in patients with other types of infections (intraabdominal infections, acute pyelonephritis, and primary gram-negative bacteremia) [35, 36]. In terms of sepsis, ACs were high in bacteremic patients that did not survive sepsis [37] and in patients with sepsis compared to non-infectious SIRS [38]. These studies show that ACs could be specific biomarkers for CAP and might be associated with disease severity as these compounds increase in non-survivors. In the current study, we also showed increased ACs in CAP non-survivors vs. survivors.

Decreased PC concentrations in blood have been reported in some invasive bacterial infectious diseases such as sepsis, CAP, and bacteremia [39]. It has been reported that there is a decreased level of PCs and phosphatidylinositol (PI) in BALF and a significant decrease in phosphatidylglycerol (PG) and SMs in severe pneumonia, and in ARDS associated with pneumonia compared to other less severe pneumonia patients and controls [40]. There is also a report of decreased level of PCaaC34:3 in CAP patients compared to different types of infections [41]. Glycerophospholipids such as lysoPCs, lysoPEs, and lysoPIs are other possible biomarkers for the diagnosis and prognosis of CAP reported in several studies, which are briefly discussed below. A decrease of lysoPEs and lysoPCs was found in CAP patients compared to non-CAP patients. LysoPEs were found decreased in fatal cases of CAP compared to non-fatal cases [36] and also were found increased in survivors of CAP compared to other types of infection [41]. Specifically, lysoPCs were significantly lower in CAP non-survivors than in survivors, introducing these as potential prognostic markers for CAP patients who require hospitalization [35].

Although similar to ACs, lysoPCs have been shown to be increased in patients with CAP compared to patients with other types of infection [41]. Nonetheless, the decreased lysoPCs (LPCs 16:0, 16:1, and 18:0) were associated with severe septic shock non-survivors at day 1 and day 7 in 28-day and 90-day mortality studies [34]. At the clinical phenotypic level, lower concentrations of lysoPCs were in inverse correlation with PSI and CURB scores in CAP non-survivors on day 1 of admission to hospital and non-survivors admitted to the ICU with severe sepsis or septic shock in 28-day mortality studies [35, 42]. The data showed glycerophospholipids, particularly lysoPCs, appear to be specific biomarkers for bacterial CAP and are probably associated with the severity of CAP as they decrease in non-survivors versus survivors. These results also support our current findings showing lower levels of lysoPCs in CAP non-survivors vs. survivors. Decreased lysoPCs are associated with the acute stage of CAP that can increase over time during treatment [27]. The data highlight the role of glycerophospholipids (lysoPCs) in respiratory diseases as lysoPCs are major lung surfactant phospholipids which are potentially involved in cellular inflammation and proliferation mechanism association with atherosclerosis and inflammatory disorders [38]. LysoPCs are the main degradation product of phospholipids when they are oxidized during apoptosis and lead to either harmless or highly toxic phospholipids [43, 44].

Regardless of the role of lipid profiling for the diagnosis and prognosis of bacterial CAP, profiling of other metabolites has been widely applied to diagnose and differentiate respiratory disorders including CAP. Plasma-based metabolomics of 240 critically ill patients (SIRS, sepsis, sepsis-induced ARDS) revealed the application of metabolomic profiling for the prognosis of 28-day mortality using GC-MS and LC-MS techniques. Amino acids, carbohydrates, and nucleotides were among the most differentiating metabolites to predict the mortality [45]. In a study of only 30 CAP patients from the GenIMS study, the same study population of the current study, UHPLC-MS/MS- and GC-MS-based metabolomics showed a contribution of 423 metabolites to separate survivor from non-survivor cohorts. Of these, 56 metabolites were selected based on their lower false discovery rate (*q* < 0.1) which showed the biggest differences between the two cohorts [46]. Also, increased phytosphingosine, sphinganine, creatine, lactate, and methoxyacetic acid and decreased 4-hydroxybenzensulfonic acid, dehydroepiandrosterone sulfate (DHEA-S), and l-arginine were capable of differentiating patients with severe CAP from a non-severe cohort [47]. The high sensitivity of metabolites to intrinsic stimuli in association with high throughput revealed 11 volatile organic compounds (VOCs) in exhaled breath samples which could discriminate pneumonia patients from controls (patients without pneumonia). Moreover, 52 VOCs were significantly lower in patients with positive cultures compared to those with negative culture [48].

Here we show that metabolites, particularly lipids, could be more reflective biomarkers for prognosis of mortality of bacterial CAP rather than other historic biomarkers such as proteins and cytokines. Abnormally expressed plasma cytokines, chemokines, and PCT and CRP can be used for the diagnosis and outcome prediction of CAP; however, they may not be predictive enough for the prognosis of CAP outcomes especially early in the disease process [6]. Also, lipid profiling particularly ACs and lysoPCs may be considered as further potential biomarker to assess patients who need ICU admission and to assess pneumonia severity. In addition, the most common severity scoring systems do not have high enough AUROCs to be capable of predicting 30-day mortality in CAP [8].

PCs and lysoPCs are the most abundant glycerophospholipids and are major components of all cell membranes and pulmonary surfactant [49], and moreover, PCs are the most abundant phospholipids contributing to ATP synthesis and multiple critical mitochondrial functions such as apoptosis, autophagy, and mitochondrial electron transport chain reaction [50]. Importantly, the decrease of PCs and lysoPCs could reflect a loss of alveolar epithelial cells and their functions.

The strength of the current study is reflected by the comprehensive metabolomic approach that shows the potential application of lipid profiling for the prognosis of 90-day mortality and in-hospital mortality based on samples from the 1st day of admission to hospital using highly specific and significant predictive models. Although the prediction score (*Q*^2^ = 0.298) may not be high for the prognosis of 90-day mortality in this study, the intercorrelation of lipid compounds from the same subclass (i.e., lysoPCs, SMs, PCs, and ACs) and similarly changing trends of lipid metabolites enhances the predicting power of lipid profiling for the prognosis of mortality. Additionally, using both multivariate and univariate data analysis, two different approaches, showed the same changing trends among lipids, which strengthens the probability of the prediction value of lipids in bacterial CAP.

Limitations of this study include a relatively small sample size especially since there is considerable heterogeneity of the bacterial CAP cohorts due to comorbidities such as sepsis, CHF, and neurological disorders (Table 1) and the heterogeneity within complexity and severity of bacterial CAP can affect the prediction power. In addition, the prediction power for the prognosis of short-term outcome may not be as strong in retrospective case-control studies when compared to prospective studies. Adding to the variation of the study, sampling has been done in more than 24 centers (in this multicenter study population). Sample handling in multicenter studies could be one of the major sources of variation among samples impacting metabolomic profiling and therefore prediction accuracy. Nonetheless, we believe that the multicenter sampling, in particular, the geographical distribution of the current study population might be a potential strength for the validity of prognosis of mortality in which samples represent the different populations of north, northwest, and central USA. Validation of this study using lipid-based metabolomic determination is required in further analyses.

## Conclusion

Targeted lipid profiling using a relatively simple DI-MS/MS method can be used for the prognosis of 90-day mortality and in-hospital mortality and can help determine the need for ICU admission and help assess severity among patients with bacterial CAP in a clinical setting. This study requires validation using an independent cohort of patients. We believe that lipid metabolomics can enhance current prognostic tests and can be a useful addition to biomarkers such as inflammatory cytokines, PCT, CRP, and severity scoring systems to predict outcomes in CAP patients admitted to the hospital.

## Supplementary information

**Additional file 1: Figure S1.** Coefficient plot shows a relative concentration of 20 metabolites involve in the discrimination of non-survivors from survivors by DI-MS/MS. **Table S1.** Unpaired t-tests show 32 metabolites with significant changes [(FDR < 0.05), highlighted in blue] between in-hospital deaths and survivors detected in plasma using DI-MS/MS. **Figure S2.** Permutation test (200 times) of OPLS-DA model of metabolites in plasma obtained by DI-MS/MS, to validate the predictability of the model to separate survivors (*n* = 75) from non-survivors (n = 75). The test shows the prediction is valid. **Figure S3.** PLS-regression shows a very strong relationship between the most differentiating metabolites (*n* = 20) in the separation of survivors and non-survivors in plasma detected by DI-MS/MS. **Figure S4.** Coefficient plot shows the relative concentration of increased and decreased metabolites in the in-hospital mortality versus survivors (> 90 days) based on the DI-MS/MS data. **Table S2.** Unpaired t-test shows 65 metabolites significantly changed [(FDR < 0.05, highlighted in blue) between in-hospital deaths and survivors (> 90 days) detected by DI-MS/MS of plasma. **Figure S6**, PLS-regression analysis shows a very strong correlation between most differentiating metabolites separating survivors from in-hospital deaths (using the most differentiating metabolites, *n* = 22) detected by DI-MS/MS of plasma. **Figure S7.** Permutation test (200 times) to validate the predictability of the model to separate in hospital deaths (*n* = 26) from survivors (*n* = 75). The test shows the OPLS-DA prediction is valid. **Figure S8.** Partial least square regression (PLSR) analysis shows that the most differentiating metabolites obtained by DI-MS/MS are in strong relationship with APACHE III and PSI scores, showing metabolites are highly correlated with paraclinical features. A: APACHE III, B: PSI at day 0, C: PSI at day 1, and: PSI no Age. **Table S3.** Logistic regression of APACHE III and PSI to predict mortality. This table shows that severity scoring systems (APACHE III and PSI) are not as good as metabolites biomarkers for the prediction of mortality in these cohorts because they have lower sensitivity, specificity, AUROC, *p* values and regression compared to metabolomics lipid profiling. **Table S4**. Characteristics of bacterial CAP ICU patients vs. ICU ventilated controls. ŧ APACHE III, ŧ ŧ APACHE II. **Figure S9.** OPLS-DA of NMR metabolites show a very predictable model to separate the bacterial CAP ICU patients (*n* = 41) from ICU ventilated control (*n* = 31) using ^1^H-NMR, R^2^Y = 0.871, Q^2^Y = 0.777, and *p* = 1.97× 10^− 21^. **Figure S10.** OPLS-DA of GC-MS features show an excellent prediction model to separate the bacterial CAP ICU patients (n = 41) from ICU ventilated control (n = 31) using GC-MS, R^2^Y = 0.915, Q^2^Y = 0.852, and *p* = 1.3× 10^− 16^. **Table S5.** Summarized OPLS-DA models of two platform to separate ICU-admitted CAP pneumonia (*n* = 41) from ICU ventilated controls (*n* = 31), Both NMR and GC-MS show an excellent prediction models as verified by Q^2^Y, p value, sensitivity, specificity and AUROC parameters. (AUROC = area under receiver operating curve). **Table S6.** Unpaired t-test shows 22 known metabolites obtained by DI-MS/MS significantly changed (*p* < 0.05, while 13 metabolites highlighted had an FDR < 0.05) between ICU and non-ICU CAP patients. **Table S7.** Unpaired t-test shows 37 known features significantly changed (p < 0.05, while 12 metabolites highlighted had a FDR < 0.05) between patients with severe pneumonia (PSI 4 and 5) vs. patients with less severe pneumonia (PSI 1, 2 and 3). **Figure S11.** Heatmap analysis shows a separation between patients with more severe (PSI 4 and 5) and less severe (PSI 1, 2 and 3) CAP using the most differentiating metabolites by DI-MS/MS. **Table S8.** Unpaired t-test shows 27 metabolites obtained by NMR significantly changed (highlighted FDR < 0.05) between ICU-admitted bacterial CAP and ICU ventilated controls. **Table S9.** Unpaired t-test shows 46 known features obtained by GC-MS significantly changed (FDR < 0.05) between ICU-admitted bacterial CAP and ICU ventilated controls. **Table S10.** Comparison of three analytical platforms shows that DI-MS/MS is more predictive and significant for 90 day mortality than GC-MS and NMR. **Table S11.** The characteristics of CAP patients who were admitted to ICU vs. CAP patients who were not admitted to ICU.

## Data Availability

Data are available on request.

## Abbreviations

AC: Acylcarnitine
APACHE: Acute Physiology and Chronic Health Evaluation
ARDS: Acute respiratory distress syndrome
BALF: Bronchoalveolar lavage fluid
AUROC: Area under the receiver operating curve
^1^H-NMR: Proton nuclear magnetic resonance
GC-MS: Gas chromatography mass spectrometry
ICU: Intensive care unit
CAP: Community-acquired pneumonia
CHF: Cardiac heart failure
CRP: C-reactive protein
DI-MS/MS: Direct infusion tandem mass spectrometry
FDR: False discovery rate
GC-TOF-MS: Gas chromatography time of flight mass spectrometry
*m*/*z*: Mass to charge ratio
LOS: Length of stay
NIST: National Institute of Standards and Technology
PCA: Principal component analysis
PCT: Procalcitonin
PSI: Pneumonia severity index
OPLS-DA: Orthogonal partial least-squares discriminant analysis
CV-ANOVA: Cross-validation-analysis of variance
LysoPC: Lysophosphatidylcholine
LysoPE: Lysophophatidylethanolamine
PC: Phosphatidylcholine
PLSR: Partial least square regression
SAPS: Simplified Acute Physiology Score
SM: Sphingomyelin
SOFA: Sequential Organ Failure Assessment
MVA: Multivariate data analysis
UVA: Univariate data analysis
VIP: Variable important in projection
VOC: Volatile organic compound
