# Supplementary material for: Plasma lipid profiling for the prognosis of 90-day mortality, in-hospital mortality, ICU admission, and severity in bacterial community-acquired pneumonia (CAP)
Source: Crit Care. 2020 Jul 27;24:461. doi: 10.1186/s13054-020-03147-3 (PMC7385943; doi:10.1186/s13054-020-03147-3)
Supplement: Supplementary file 1 — Additional file 1: Figure S1. Coefficient plot shows a relative concentration of 20 metabolites involve in the discrimination of non-survivors from survivors by DI-MS/MS. Table S1. Unpaired t-tests show 32 metabolites with significant changes [(FDR < 0.05), highlighted in blue] between in-hospital deaths and survivors detected in plasma using DI-MS/MS. Figure S2. Permutation test (200 times) of OPLS-DA model of metabolites in plasma obtained by DI-MS/MS, to validate the predictability of the model to separate survivors (n = 75) from non-survivors (n = 75). The test shows the prediction is valid. Figure S3. PLS-regression shows a very strong relationship between the most differentiating metabolites (n = 20) in the separation of survivors and non-survivors in plasma detected by DI-MS/MS. Figure S4. Coefficient plot shows the relative concentration of increased and decreased metabolites in the in-hospital mortality versus survivors (> 90 days) based on the DI-MS/MS data. Table S2. Unpaired t-test shows 65 metabolites significantly changed [(FDR < 0.05, highlighted in blue) between in-hospital deaths and survivors (> 90 days) detected by DI-MS/MS of plasma. Figure S6, PLS-regression analysis shows a very strong correlation between most differentiating metabolites separating survivors from in-hospital deaths (using the most differentiating metabolites, n = 22) detected by DI-MS/MS of plasma. Figure S7. Permutation test (200 times) to validate the predictability of the model to separate in hospital deaths (n = 26) from survivors (n = 75). The test shows the OPLS-DA prediction is valid. Figure S8. Partial least square regression (PLSR) analysis shows that the most differentiating metabolites obtained by DI-MS/MS are in strong relationship with APACHE III and PSI scores, showing metabolites are highly correlated with paraclinical features. A: APACHE III, B: PSI at day 0, C: PSI at day 1, and: PSI no Age. Table S3. Logistic regression of APACHE III and PSI to predict mortali [file 13054_2020_3147_MOESM1_ESM.docx]

**Plasma lipid profiling for the prognosis of 90-day mortality, in-hospital mortality, ICU admission and severity in bacterial community acquired pneumonia (CAP)**

Mohammad M. Banoei^1^, Hans J. Vogel^2^, Aalim M. Weljie^2,3^, Sachin Yende^4,5^, Derek C. Angus^4,5^ and Brent W. Winston^1, 6^

^1^ Department of Critical Care Medicine, Faculty of Medicine, University of Calgary, Alberta, Canada.

^2^ Department of Biological Sciences, University of Calgary, Calgary, Alberta, Canada.

^3^ Department of Pharmacology, University of Pennsylvania, Philadelphia, Pennsylvania, USA.

^4^ The Clinical Research, Investigation, and Systems Modeling of Acute Illness (CRISMA) Laboratory, University of Pittsburgh, Pittsburgh, PA, USA.

^5^Department of Critical Care Medicine, University of Pittsburgh, Pittsburgh, PA, USA. ^6^ Departments of Medicine and Biochemistry and Molecular Biology, University of Calgary, Calgary, Alberta, Canada.

**Address correspondence to:**

Brent W. Winston, MD, Departments of Critical Care Medicine, Medicine and Biochemistry and Molecular Biology, University of Calgary, Health Research Innovation Center (HRIC), Room 4C64, 3280 Hospital Drive N.W., Calgary, Alberta, Canada, T2N 4Z6.

Tel: (403) 220-4331

Fax: (403) 283-1267

Email: [bwinston@ucalgary.ca](mailto:bwinston@ucalgary.ca)

**Supplementary Figures and Tables**

## Direct infusion tandem mass spectrometry (DI-MS/MS)

### Samples preparation

Plasma samples were centrifuged at 13,000 x g. 10µl of plasma sample was filtered and placed in a well of 96-well plate. 20 µL of a 5% solution of phenyl isocyanate was added to each sample for derivatization. The filter spots were dried using an evaporator after incubation. 300 µL of methanol containing 5 mM ammonium acetate was used to extract metabolite from the plasma sample. The extracts were centrifuged into a lower 96-deep well plate, followed by a dilution step with kit MS running solvent.

### DI-MS/MS analysis and data acquisition

To quantify the metabolites in the plasma samples, we used direct injection mass spectrometry with a 96 well Absolute *IDQ*™ Kit (BIOCRATES Life Sciences AG, Innsbruck, Austria). The kit was purchased from BIOCRATES Life Sciences AG (Austria). We used an ABI 4000 Q-Trap tandem mass spectrometry instrument (Applied Biosystems/MDS Analytical Technologies, Foster City, CA) equipped with a solvent delivery system for the targeted identification and quantification 162 metabolites including amino acids, acylcarnitines, biogenic amines, glycerophospholipids, sphingolipids and sugars. For absolute quantification, isotope-labeled internal standards and other internal standards are integrated in the kit plate filter. All the samples were analyzed using the protocol described in the AbsoluteIDQ user manual. The samples were delivered to the mass spectrometer by a direct injection (DI) method.

### DI-MS/MS data acquisition and processing

We used Biocrates MetIQ software to control the entire assay workflow, from sample registration to automated calculation of metabolite concentrations to the export of data for data analysis. A targeted profiling scheme was used to quantitatively screen for known small molecule metabolites using multiple reaction monitoring, neutral loss and precursor ion scans.


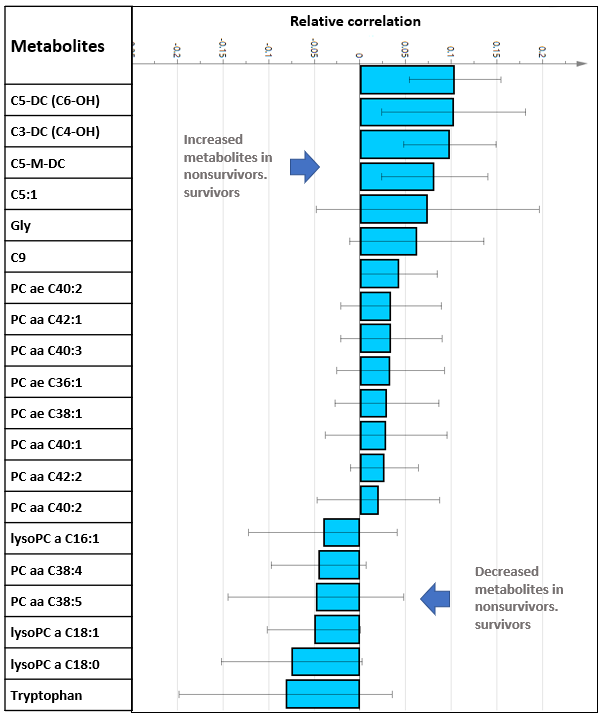


**Figure S1**. Coefficient plot shows a relative concentration of 20 metabolites involve in the discrimination of non-survivors from survivors by DI-MS/MS.

|  | **Name** | **Mean (SD) of Survivors** | **Mean (SD) of Non-survivors** | **p-value** | **q-value (FDR)** | **Fold Change** | **non-survivors/survivors** |
| --- | --- | --- | --- | --- | --- | --- | --- |
| **1** | **Tryptophan** | 49.916 (13.539) | 40.527 (12.101) | < 0.0001 | 0.0009 | 1.23 | Down |
| **2** | **lysoPC a C18:0** | 14.946 (5.166) | 11.289 (5.063) | < 0.0001 | 0.0009 | 1.32 | Down |
| **3** | **C5-DC C6-OH** | 0.018 (0.007) | 0.027 (0.016) | < 0.0001 (W) | 0.0009 | -1.48 | Up |
| **4** | **C3-DC C4-OH** | 0.058 (0.032) | 0.081 (0.057) | 0.0002 (W) | 0.0022 | -1.4 | Up |
| **5** | **C9** | 0.028 (0.013) | 0.038 (0.020) | 0.0005 (W) | 0.0041 | -1.35 | Up |
| **6** | **C5-M-DC** | 0.026 (0.008) | 0.038 (0.025) | 0.0008 (W) | 0.0043 | -1.44 | Up |
| **7** | **lysoPC a C16:1** | 1.772 (0.694) | 1.419 (0.752) | 0.0008 (W) | 0.0043 | 1.25 | Down |
| **8** | **PC aa C38:5** | 61.782 (18.528) | 50.879 (20.534) | 0.0009 (W) | 0.0043 | 1.21 | Down |
| **9** | **PC aa C38:4** | 123.740 (36.909) | 102.975 (39.756) | 0.0011 (W) | 0.0048 | 1.2 | Down |
| **10** | **lysoPC a C16:0** | 50.749 (16.900) | 41.016 (19.607) | 0.0014 (W) | 0.0055 | 1.24 | Down |
| **11** | **lysoPC a C20:3** | 1.251 (0.673) | 0.934 (0.626) | 0.0017 (W) | 0.006 | 1.34 | Down |
| **12** | **lysoPC a C18:2** | 12.896 (7.451) | 9.482 (6.505) | 0.0018 (W) | 0.006 | 1.36 | Down |
| **13** | **PC aa C40:6** | 21.351 (8.042) | 17.429 (7.733) | 0.0024 (W) | 0.0073 | 1.23 | Down |
| **14** | **PC ae C38:1** | 1.325 (1.263) | 1.992 (2.067) | 0.0033 (W) | 0.0088 | -1.5 | Up |
| **15** | **lysoPC a C18:1** | 12.745 (5.220) | 10.195 (4.802) | 0.0033 (W) | 0.0088 | 1.25 | Down |
| **16** | **lysoPC a C20:4** | 4.527 (2.157) | 3.594 (2.180) | 0.0038 (W) | 0.0094 | 1.26 | Down |
| **17** | **PC aa C38:3** | 45.728 (14.803) | 38.671 (13.738) | 0.0040 (W) | 0.0094 | 1.18 | Down |
| **18** | **PC aa C40:5** | 10.067 (3.146) | 8.764 (3.199) | 0.0053 (W) | 0.0118 | 1.15 | Down |
| **19** | **SM C22:3** | 1.726 (0.585) | 1.527 (0.762) | 0.0070 (W) | 0.0147 | 1.13 | Down |
| **20** | **PC aa C36:3** | 145.348 (43.733) | 125.794 (38.652) | 0.0076 (W) | 0.0148 | 1.16 | Down |
| **21** | **PC aa C38:6** | 60.389 (19.821) | 52.101 (24.033) | 0.0078 (W) | 0.0148 | 1.16 | Down |
| **22** | **PC aa C40:2** | 0.424 (0.352) | 0.623 (0.661) | 0.0102 (W) | 0.0186 | -1.47 | Up |
| **23** | **PC ae C36:4** | 15.308 (3.955) | 13.737 (4.488) | 0.0144 (W) | 0.0244 | 1.11 | Down |
| **24** | **SM OH C22:1** | 11.128 (3.274) | 9.628 (3.705) | 0.0146 (W) | 0.0244 | 1.16 | Down |
| **25** | **PC ae C40:3** | 2.114 (1.634) | 2.891 (2.331) | 0.0182 (W) | 0.0284 | -1.37 | Up |
| **26** | **PC ae C40:0** | 7.397 (3.034) | 6.226 (2.380) | 0.0185 (W) | 0.0284 | 1.19 | Down |
| **27** | **PC aa C36:2** | 220.127 (58.441) | 196.960 (54.998) | 0.0206 (W) | 0.0305 | 1.12 | Down |
| **28** | **PC aa C36:4** | 187.139 (44.828) | 170.039 (71.722) | 0.0213 (W) | 0.0305 | 1.1 | Down |
| **29** | **lysoPC a C17:0** | 0.925 (0.321) | 0.776 (0.316) | 0.0251 (W) | 0.0347 | 1.19 | Down |
| **30** | **PC aa C40:3** | 0.614 (0.317) | 0.782 (0.542) | 0.0292 (W) | 0.0381 | -1.27 | Up |
| **31** | **PC ae C36:1** | 9.158 (3.740) | 10.852 (5.356) | 0.0295 (W) | 0.0381 | -1.19 | Up |
| **32** | **PC ae C38:4** | 15.476 (4.045) | 14.083 (4.370) | 0.0399 (W) | 0.0499 | 1.1 | Down |
| **33** | **PC aa C32:3** | 0.943 (0.323) | 0.871 (0.459) | 0.0464 (W) | 0.0546 | 1.08 | Down |
| **34** | **PC aa C34:4** | 1.295 (0.559) | 1.170 (0.816) | 0.0464 (W) | 0.0546 | 1.11 | Down |
| **35** | **PC aa C36:5** | 15.284 (5.052) | 13.932 (7.789) | 0.0500 (W) | 0.0571 | 1.1 | Down |
| **36** | **PC aa C42:2** | 0.221 (0.086) | 0.263 (0.140) | 0.0543 (W) | 0.0604 | -1.19 | Up |
| **37** | **PC aa C42:4** | 0.252 (0.141) | 0.319 (0.218) | 0.0560 (W) | 0.0606 | -1.27 | Up |
| **38** | **PC ae C40:2** | 1.749 (0.634) | 1.992 (0.812) | 0.0646 (W) | 0.068 | -1.14 | Up |
| **39** | **PC aa C40:1** | 0.423 (0.146) | 0.493 (0.239) | 0.0693 (W) | 0.0711 | -1.17 | Up |
| **40** | **PC ae C38:2** | 2.628 (1.590) | 3.485 (2.807) | 0.0721 (W) | 0.0721 | -1.33 | Up |

**Table S1.** Unpaired t-tests show 32 metabolites with significant changes [(FDR< 0.05), highlighted in blue] between in-hospital deaths and survivors detected in plasma using DI-MS/MS.


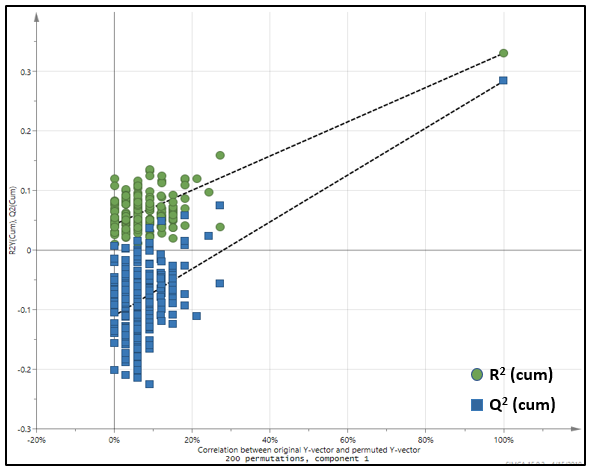


**Figure S2**. Permutation test (200 times) of OPLS-DA model of metabolites in plasma obtained by DI-MS/MS, to validate the predictability of the model to separate survivors (n=75) from non-survivors (n=75). The test shows the prediction is valid.


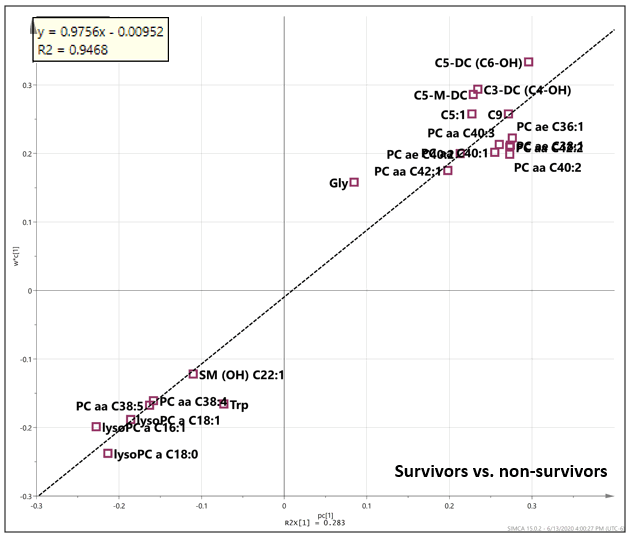


**Figure S3**. PLS-regression shows a very strong relationship between the most differentiating metabolites (n=20) in the separation of survivors and non-survivors in plasma detected by DI-MS/MS.


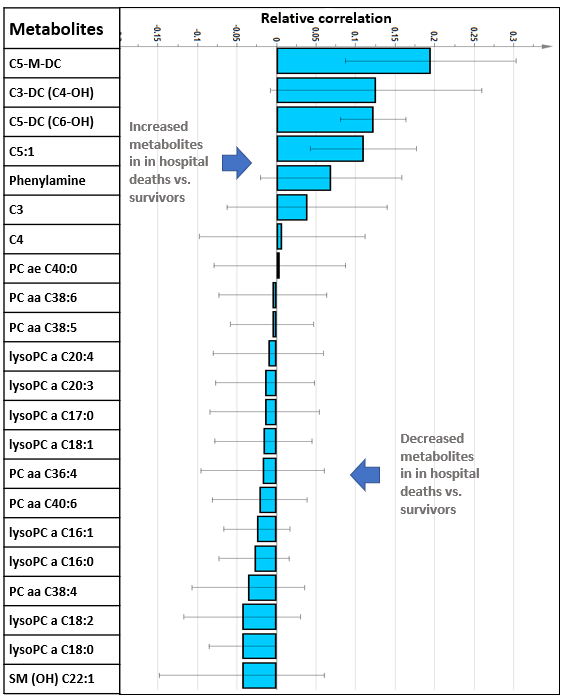


**Figure S4**. Coefficient plot shows the relative concentration of increased and decreased metabolites in the in-hospital mortality versus survivors (>90 days) based on the DI-MS/MS data.

|  | **Name** | **Mean (SD) of Survivor** | **Mean (SD) of in hospital death** | **p-value** | **q-value (FDR)** | **Fold Change** | **In hospital death /survivors** |
| --- | --- | --- | --- | --- | --- | --- | --- |
| 1 | C10:2 | 0.046 (0.023) | 0.065 (0.039) | 0.0040 (W) | 0.013 | -1.4 | Up |
| 2 | C14:1 | 0.108 (0.047) | 0.078 (0.048) | 0.0018 (W) | 0.0075 | 1.37 | Down |
| 3 | C14:2 | 0.043 (0.024) | 0.033 (0.021) | 0.0190 (W) | 0.0475 | 1.3 | Down |
| 4 | C16 | 0.087 (0.029) | 0.069 (0.029) | 0.0049 (W) | 0.0155 | 1.26 | Down |
| 5 | C18 | 0.038 (0.014) | 0.028 (0.012) | 0.0003 (W) | 0.0019 | 1.37 | Down |
| 6 | C18:1 | 0.169 (0.067) | 0.140 (0.065) | 0.0164 (W) | 0.0424 | 1.21 | Down |
| 7 | C2 | 8.559 (3.903) | 11.312 (5.756) | 0.0203 (W) | 0.049 | -1.32 | Up |
| 8 | C3 | 0.314 (0.136) | 0.486 (0.326) | 0.0157 (W) | 0.0413 | -1.55 | Up |
| 9 | C3-DC C4-OH | 0.059 (0.032) | 0.103 (0.077) | 0.0003 (W) | 0.0018 | -1.75 | Up |
| 10 | C4 | 0.261 (0.180) | 0.470 (0.354) | 0.0129 (W) | 0.0358 | -1.8 | Up |
| 11 | C5:1 | 0.023 (0.008) | 0.042 (0.032) | 0.0002 (W) | 0.0012 | -1.86 | Up |
| 12 | C5:1-DC | 0.013 (0.005) | 0.017 (0.007) | 0.0113 (W) | 0.0319 | -1.31 | Up |
| 13 | C5-DC C6-OH | 0.019 (0.008) | 0.030 (0.014) | 0.0001 (W) | 0.0008 | -1.6 | Up |
| 14 | C5-M-DC | 0.027 (0.010) | 0.045 (0.031) | 0.0012 (W) | 0.0052 | -1.66 | Up |
| 15 | C9 | 0.030 (0.015) | 0.042 (0.021) | 0.0062 (W) | 0.0185 | -1.39 | Up |
| 16 | lysoPC a C16:0 | 49.654 (17.227) | 29.989 (17.388) | < 0.0001 (W) | 0 | 1.66 | Down |
| 17 | lysoPC a C16:1 | 1.727 (0.701) | 1.107 (0.704) | < 0.0001 (W) | 0 | 1.56 | Down |
| 18 | lysoPC a C17:0 | 0.911 (0.318) | 0.584 (0.299) | < 0.0001 (W) | 0.0002 | 1.56 | Down |
| 19 | lysoPC a C18:0 | 14.575 (5.280) | 8.188 (4.484) | < 0.0001 (W) | 0 | 1.78 | Down |
| 20 | lysoPC a C18:1 | 12.439 (5.248) | 7.291 (3.041) | < 0.0001 (W) | 0 | 1.71 | Down |
| 21 | lysoPC a C18:2 | 12.523 (7.428) | 5.710 (3.307) | < 0.0001 (W) | 0 | 2.19 | Down |
| 22 | lysoPC a C20:3 | 1.221 (0.665) | 0.678 (0.468) | < 0.0001 (W) | 0.0002 | 1.8 | Down |
| 23 | lysoPC a C20:4 | 4.373 (2.196) | 2.242 (1.414) | < 0.0001 (W) | 0 | 1.95 | Down |
| 24 | PC aa C32:3 | 0.938 (0.317) | 0.691 (0.371) | 0.0027 (W) | 0.0095 | 1.36 | Down |
| 25 | PC aa C34:2 | 409.328 (95.539) | 360.445 (102.600) | 0.0286 | 0.0631 | 1.14 | Down |
| 26 | PC aa C34:4 | 1.272 (0.556) | 1.097 (1.193) | 0.0033 (W) | 0.0114 | 1.16 | Down |
| 27 | PC aa C36:2 | 220.899 (58.481) | 178.837 (58.207) | 0.0019 (W) | 0.0075 | 1.24 | Down |
| 28 | PC aa C36:3 | 144.789 (42.958) | 111.013 (40.636) | 0.0007 (W) | 0.0033 | 1.3 | Down |
| 29 | PC aa C36:4 | 184.149 (45.511) | 137.178 (66.107) | < 0.0001 (W) | 0.0006 | 1.34 | Down |
| 30 | PC aa C36:5 | 14.962 (5.091) | 12.183 (10.286) | 0.0038 (W) | 0.0126 | 1.23 | Down |
| 31 | PC aa C38:0 | 3.054 (1.052) | 2.270 (0.798) | 0.0009 (W) | 0.0041 | 1.35 | Down |
| 32 | PC aa C38:3 | 45.244 (14.607) | 33.674 (15.878) | 0.0001 (W) | 0.0008 | 1.34 | Down |
| 33 | PC aa C38:4 | 121.258 (37.445) | 84.463 (37.818) | < 0.0001 (W) | 0.0002 | 1.44 | Down |
| 34 | PC aa C38:5 | 60.523 (18.776) | 42.625 (19.894) | < 0.0001 (W) | 0.0004 | 1.42 | Down |
| 35 | PC aa C38:6 | 59.258 (20.065) | 38.840 (18.934) | < 0.0001 (W) | 0.0002 | 1.53 | Down |
| 36 | PC aa C40:4 | 3.613 (1.119) | 3.147 (1.729) | 0.0036 (W) | 0.0123 | 1.15 | Down |
| 37 | PC aa C40:5 | 9.915 (3.126) | 8.008 (4.016) | 0.0012 (W) | 0.0052 | 1.24 | Down |
| 38 | PC aa C40:6 | 20.910 (8.051) | 13.472 (6.446) | < 0.0001 (W) | 0.0002 | 1.55 | Down |
| 39 | PC aa C42:0 | 0.558 (0.173) | 0.472 (0.186) | 0.0339 | 0.0696 | 1.18 | Down |
| 40 | PC ae C34:2 | 10.057 (3.260) | 8.265 (3.199) | 0.0138 (W) | 0.0376 | 1.22 | Down |
| 41 | PC ae C34:3 | 5.556 (2.433) | 4.351 (2.552) | 0.0167 (W) | 0.0426 | 1.28 | Down |
| 42 | PC ae C36:3 | 7.668 (2.511) | 6.127 (2.511) | 0.0052 (W) | 0.0163 | 1.25 | Down |
| 43 | PC ae C36:4 | 15.085 (3.964) | 11.328 (3.697) | < 0.0001 | 0.0004 | 1.33 | Down |
| 44 | PC ae C36:5 | 8.753 (2.807) | 6.368 (2.954) | 0.0005 (W) | 0.0024 | 1.37 | Down |
| 45 | PC ae C38:0 | 1.792 (0.759) | 1.468 (1.128) | 0.0058 (W) | 0.0176 | 1.22 | Down |
| 46 | PC ae C38:4 | 15.259 (4.050) | 11.392 (3.982) | < 0.0001 (W) | 0.0008 | 1.34 | Down |
| 47 | PC ae C38:5 | 20.646 (5.779) | 15.895 (5.531) | 0.0006 (W) | 0.0031 | 1.3 | Down |
| 48 | PC ae C38:6 | 6.966 (1.963) | 5.011 (1.897) | < 0.0001 | 0.0002 | 1.39 | Down |
| 49 | PC ae C40:0 | 7.256 (3.007) | 4.708 (2.042) | < 0.0001 (W) | 0.0002 | 1.54 | Down |
| 50 | PC ae C40:1 | 0.977 (0.406) | 0.774 (0.599) | 0.0022 (W) | 0.0081 | 1.26 | Down |
| 51 | PC ae C40:5 | 5.341 (1.699) | 4.561 (2.170) | 0.0154 (W) | 0.0411 | 1.17 | Down |
| 52 | PC ae C40:6 | 4.862 (1.288) | 3.561 (1.216) | < 0.0001 | 0.0002 | 1.37 | Down |
| 53 | Phenylalanine | 73.685 (23.018) | 111.656 (71.096) | 0.0011 (W) | 0.005 | -1.52 | Up |
| 54 | SM C16:1 | 15.933 (4.005) | 12.514 (4.645) | 0.0005 | 0.0024 | 1.27 | Down |
| 55 | SM C18:0 | 26.216 (6.605) | 21.107 (10.045) | 0.0025 (W) | 0.009 | 1.24 | Down |
| 56 | SM C18:1 | 15.662 (3.851) | 11.739 (6.294) | 0.0013 (W) | 0.0053 | 1.33 | Down |
| 57 | SM C20:2 | 1.261 (0.366) | 0.979 (0.455) | 0.0019 | 0.0075 | 1.29 | Down |
| 58 | SM C22:3 | 1.696 (0.585) | 1.195 (0.583) | 0.0002 | 0.0015 | 1.42 | Down |
| 59 | SM C24:0 | 16.703 (4.496) | 12.645 (5.150) | 0.0002 | 0.0014 | 1.32 | Down |
| 60 | SM C24:1 | 42.481 (10.382) | 37.303 (12.953) | 0.0419 | 0.0839 | 1.14 | Down |
| 61 | SM OH C16:1 | 3.567 (0.981) | 2.908 (1.248) | 0.0066 (W) | 0.0192 | 1.23 | Down |
| 62 | SM OH C22:1 | 11.020 (3.275) | 7.576 (3.510) | < 0.0001 (W) | 0.0004 | 1.45 | Down |
| 63 | SM OH C22:2 | 8.606 (2.347) | 6.331 (2.530) | < 0.0001 | 0.0005 | 1.36 | Down |
| 64 | SM OH C24:1 | 1.187 (0.321) | 1.005 (0.383) | 0.0195 | 0.0479 | 1.18 | Down |
| 65 | Tryptophan | 48.710 (14.077) | 40.150 (13.586) | 0.0063 (W) | 0.0186 | 1.21 | Down |

**Table S2.** Unpaired t-test shows 65 metabolites significantly changed [(FDR< 0.05, highlighted in blue) between in-hospital deaths and survivors (>90 days) detected by DI-MS/MS of plasma.


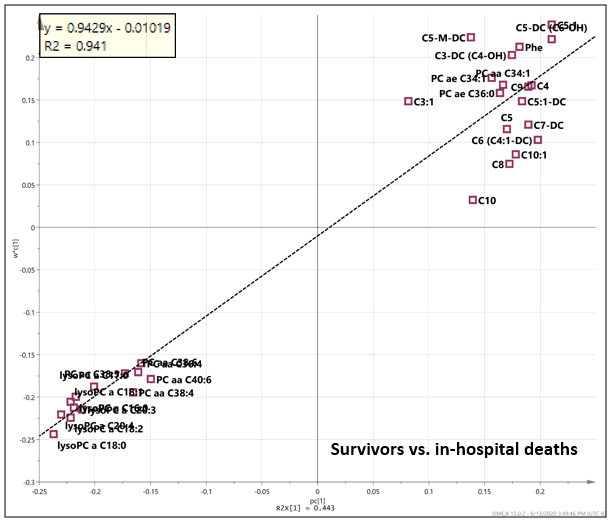


**Figure S6**, PLS-regression analysis shows a very strong correlation between most differentiating metabolites separating survivors from in-hospital deaths (using the most differentiating metabolites, n=22) detected by DI-MS/MS of plasma.


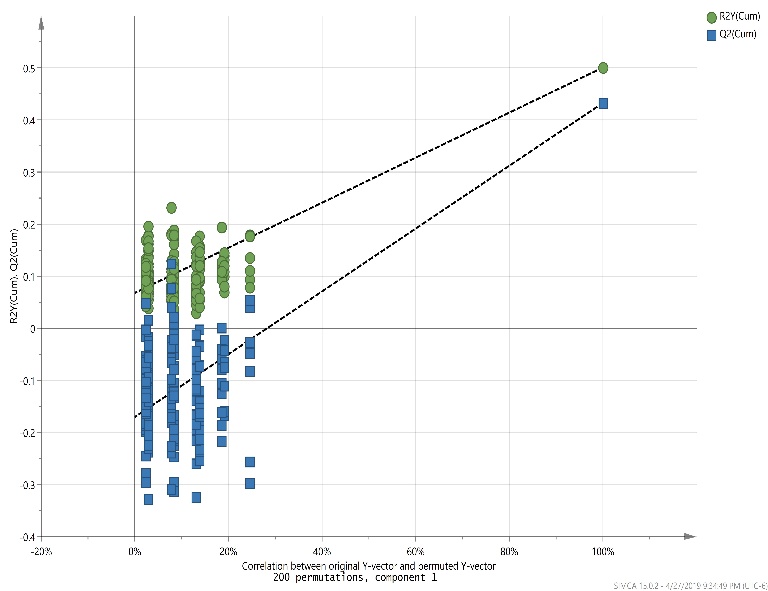


**Figure S7.** Permutation test (200 times) to validate the predictability of the model to separate in hospital deaths (n=26) from survivors (n=75). The test shows the OPLS-DA prediction is valid.


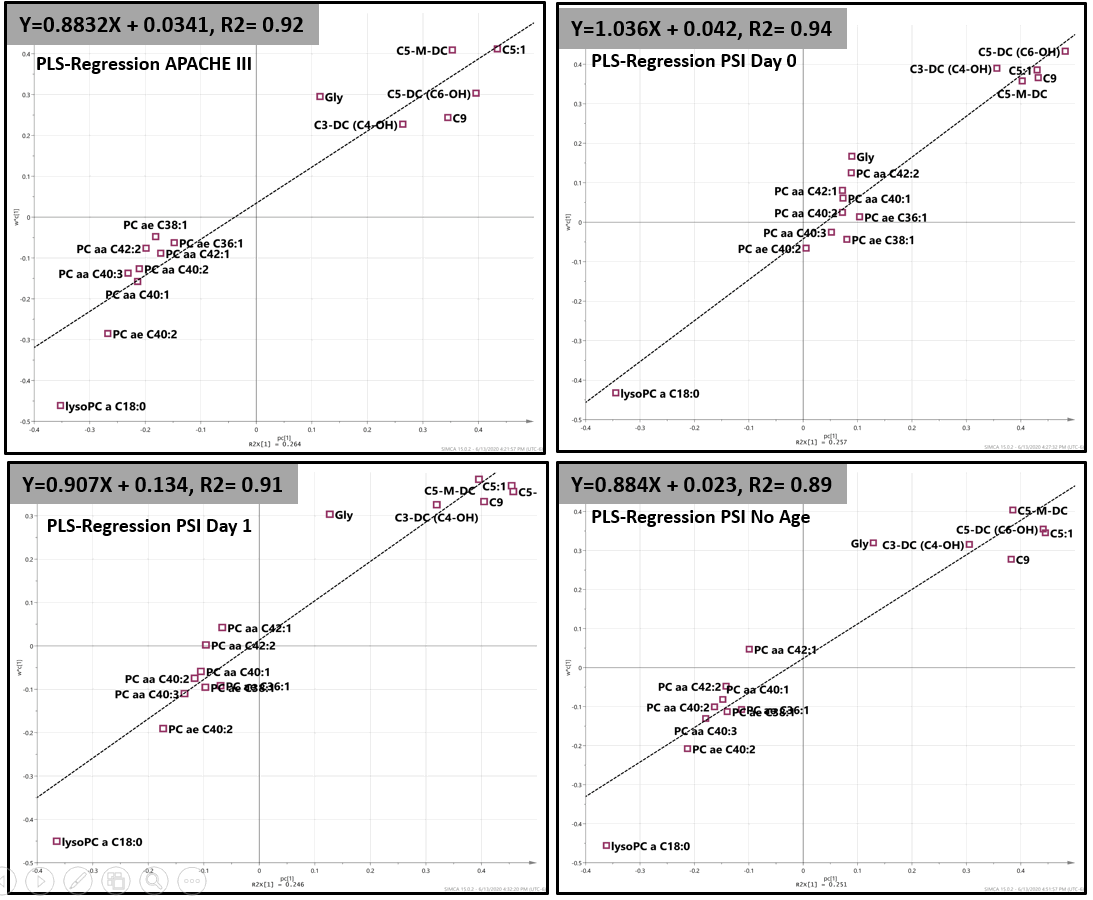


**Figure S8**. Partial least square regression (PLSR) analysis shows that the most differentiating metabolites obtained by DI-MS/MS are in strong relationship with APACHE III and PSI scores, showing metabolites are highly correlated with paraclinical features. A: APACHE III, B: PSI at day 0, C: PSI at day 1, and: PSI no Age.


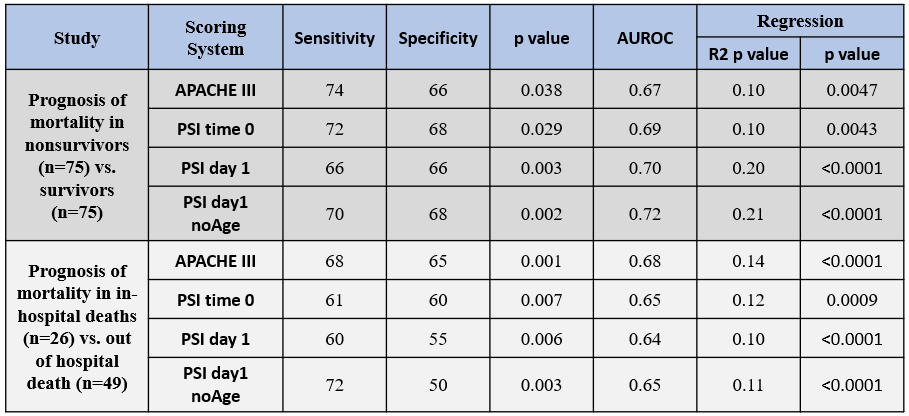


**Table S3.** Logistic regression of APACHE III and PSI to predict mortality. This table shows that severity scoring systems (APACHE III and PSI) are not as good as metabolites biomarkers for the prediction of mortality in these cohorts because they have lower sensitivity, specificity, AUROC, p values and regression compared to metabolomics lipid profiling.

| Variable | Bacterial CAP ICU Patients (n=41) | ICU Ventilated Controls (n=31) |
| --- | --- | --- |
| Age yrs. (mean ± SD) | 75.8 ± 9.5 | 50.6 ± 13.8 |
| Male/Female | 19/22 | 14/17 |
| APACHE II/ III | 77.7 ± 23.1ŧ | 19.1 ± 5.7ŧŧ |
| ICU LOS^★^ | 5.0 ± 3.8 | 4 ± 4.9 |
| Hospital LOS | 11.8 ± 8.3 | 24.7 ± 33.9 |

**Table S4**. Characteristics of bacterial CAP ICU patients vs. ICU ventilated controls. ŧ APACHE III, ŧ ŧ APACHE II


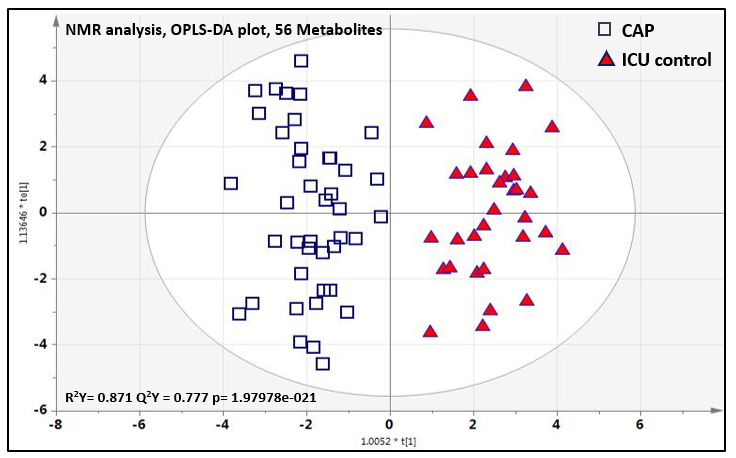


**Figure S9.** OPLS-DA of NMR metabolites show a very predictable model to separate the bacterial CAP ICU patients (n=41) from ICU ventilated control (n=31) using ^1^H-NMR, R^2^Y= 0.871, Q^2^Y= 0.777, and *p*= 1.97× 10^-21^.


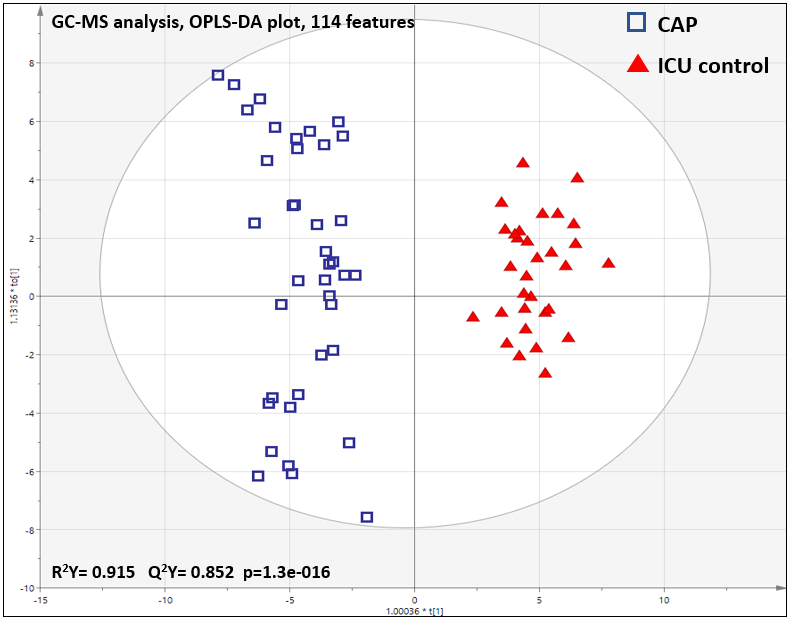


**Figure S10.** OPLS-DA of GC-MS features show an excellent prediction model to separate the bacterial CAP ICU patients (n=41) from ICU ventilated control (n=31) using GC-MS, R^2^Y= 0.915, Q^2^Y= 0.852, and *p*= 1.3× 10^-16^.

| **Analytical Tool** | **R^2^Y** | **Q^2^Y** | **P value** | **No of Metabolites** | **Sensitivity** | **Specificity** | **AUROC** |
| --- | --- | --- | --- | --- | --- | --- | --- |
| NMR | 0.871 | 0.777 | 1.97e-21 | 56 | >0.99 | >0.99 | >0.99 |
| GC-MS | 0.915 | 0.852 | 1.3e-16 | 114 | >0.99 | >0.99 | >0.99 |

Table S5. Summarized OPLS-DA models of two platform to separate ICU-admitted CAP pneumonia (n=41) from ICU ventilated controls (n=31), Both NMR and GC-MS show an excellent prediction models as verified by Q^2^Y, p value, sensitivity, specificity and AUROC parameters. (AUROC = area under receiver operating curve).

|  | **Name** | **Mean (SD) of ICU** | **Mean (SD) of Non- ICU** | **p-value** | **q-value (FDR)** | **Fold Change** | **ICU/Non ICU** |
| --- | --- | --- | --- | --- | --- | --- | --- |
| **1** | **lysoPCaC18:0** | 11.325 (6.401) | 14.962 (5.255) | 0.0084 | 0.0348 | -1.32 | Down |
| **2** | **PCaeC38:4** | 12.999 (4.273) | 15.370 (3.943) | 0.0141 | 0.0388 | -1.18 | Down |
| **3** | **SMOHC22:1** | 9.245 (3.692) | 11.108 (3.877) | 0.0353 | 0.0685 | -1.2 | Down |
| **4** | **PCaeC36:4** | 13.118 (4.461) | 15.089 (3.672) | 0.0389 | 0.0708 | -1.15 | Down |
| **5** | **lysoPCaC17:0** | 0.714 (0.396) | 0.952 (0.285) | 0.0008 (W) | 0.018 | -1.33 | Down |
| **6** | **C3-DC(C4-OH)** | 0.095 (0.068) | 0.059 (0.034) | 0.0011 (W) | 0.018 | 1.61 | Up |
| **7** | **C8:1** | 0.244 (0.116) | 0.186 (0.118) | 0.0057 (W) | 0.0348 | 1.31 | Up |
| **8** | **C5-DC (C6-OH)** | 0.029 (0.019) | 0.019 (0.007) | 0.0059 (W) | 0.0348 | 1.54 | Up |
| **9** | **C6 (C4:1-DC)** | 0.129 (0.095) | 0.080 (0.045) | 0.0067 (W) | 0.0348 | 1.6 | Up |
| **10** | **lysoPCaC20:4** | 3.405 (2.149) | 4.755 (2.239) | 0.0070 (W) | 0.0348 | -1.4 | Down |
| **11** | **PCaaC32:1** | 24.637 (12.471) | 19.464 (15.082) | 0.0074 (W) | 0.0348 | 1.27 | Up |
| **12** | **C10:2** | 0.062 (0.038) | 0.047 (0.030) | 0.0096 (W) | 0.0351 | 1.32 | Up |
| **13** | **lysoPCaC18:1** | 10.572 (6.464) | 12.717 (4.354) | 0.0108 (W) | 0.0358 | -1.2 | Down |
| **14** | **C5-M-DC** | 0.041 (0.029) | 0.027 (0.010) | 0.0138 (W) | 0.0388 | 1.5 | Up |
| **15** | **lysoPCaC18:2** | 9.991 (8.122) | 13.000 (6.586) | 0.0185 (W) | 0.0471 | -1.3 | Down |
| **16** | **lysoPCaC16:0** | 40.786 (24.297) | 49.592 (15.645) | 0.0214 (W) | 0.0504 | -1.22 | Down |
| **17** | **PCaaC38:4** | 101.879 (37.131) | 126.813 (49.964) | 0.0305 (W) | 0.0672 | -1.24 | Down |
| **18** | **C2** | 10.916 (5.288) | 8.220 (3.828) | 0.0331 (W) | 0.0682 | 1.33 | Up |
| **19** | **C8** | 0.231 (0.233) | 0.173 (0.144) | 0.0418 (W) | 0.0708 | 1.33 | Up |
| **20** | **Proline** | 119.140 (60.145) | 144.468 (64.287) | 0.0440 (W) | 0.0708 | -1.21 | Down |
| **21** | **PCaeC40:1** | 0.891 (0.483) | 1.148 (0.628) | 0.0451 (W) | 0.0708 | -1.29 | Down |
| **22** | **C5:1** | 0.038 (0.032) | 0.025 (0.012) | 0.0486 (W) | 0.0729 | 1.55 | Up |

**Table S6.** Unpaired t-test shows 22 known metabolites obtained by DI-MS/MS significantly changed (p < 0.05, while 13 metabolites highlighted had an FDR< 0.05) between ICU and non-ICU CAP patients.

|  | **Name** | **Mean (SD) of cohort with PSI grade I, II & III** | **Mean (SD) of cohort with PSI grad IV & V** | **p-value** | **q-value (FDR)** | **Fold Change** | **PSI grade I, II & III/PSI grade IV & V** |
| --- | --- | --- | --- | --- | --- | --- | --- |
| 1 | **C10:2** | 0.042 (0.020) | 0.057 (0.033) | 0.0004 (W) | 0.036 | -1.37 | Down |
| 2 | **C5-DC (C6-OH)** | 0.018 (0.007) | 0.026 (0.015) | 0.0011 (W) | 0.0398 | -1.39 | Down |
| 3 | **C5:1-DC** | 0.011 (0.004) | 0.015 (0.006) | 0.0020 (W) | 0.0398 | -1.28 | Down |
| 4 | **C7-DC** | 0.024 (0.012) | 0.032 (0.016) | 0.0021 (W) | 0.0398 | -1.3 | Down |
| 5 | **C3-DC (C4-OH)** | 0.057 (0.026) | 0.082 (0.062) | 0.0034 (W) | 0.0469 | -1.44 | Down |
| 6 | **C9** | 0.029 (0.015) | 0.037 (0.020) | 0.0051 (W) | 0.0551 | -1.27 | Down |
| 7 | **C5-M-DC** | 0.026 (0.009) | 0.035 (0.022) | 0.0070 (W) | 0.0637 | -1.33 | Down |
| 8 | **C8:1** | 0.181 (0.087) | 0.234 (0.120) | 0.0072 (W) | 0.0637 | -1.29 | Down |
| 9 | **C5:1** | 0.022 (0.008) | 0.031 (0.023) | 0.0097 (W) | 0.0765 | -1.39 | Down |
| 10 | **C14:1-OH** | 0.015 (0.004) | 0.019 (0.007) | 0.0105 (W) | 0.0788 | -1.2 | Down |
| 11 | **C10:1** | 0.202 (0.085) | 0.260 (0.170) | 0.0147 (W) | 0.1004 | -1.29 | Down |
| 12 | **C2** | 8.202 (3.924) | 10.263 (5.115) | 0.0170 (W) | 0.1061 | -1.25 | Down |
| 13 | **C6 (C41-DC)** | 0.082 (0.046) | 0.106 (0.073) | 0.0195 (W) | 0.115 | -1.29 | Down |
| 14 | **C8** | 0.170 (0.131) | 0.206 (0.174) | 0.0340 (W) | 0.153 | -1.21 | Down |
| 15 | **lysoPC a C6:0** | 0.021 (0.014) | 0.030 (0.033) | 0.0450 (W) | 0.1876 | -1.39 | Down |
| 16 | **lysoPC a C18:0** | 15.078 (5.132) | 11.930 (5.736) | 0.0006 (W) | 0.036 | 1.26 | Up |
| 17 | **PC aa C38:3** | 47.114 (15.335) | 39.008 (14.028) | 0.0007 (W) | 0.036 | 1.21 | Up |
| 18 | **SM C24:0** | 17.742 (4.388) | 15.243 (5.690) | 0.0017 (W) | 0.0398 | 1.16 | Up |
| 19 | **PC aa C40:4** | 3.901 (1.374) | 3.294 (1.040) | 0.0019 (W) | 0.0398 | 1.18 | Up |
| 20 | **SM OH C22:1** | 11.411 (3.078) | 9.637 (3.840) | 0.0031 (W) | 0.0469 | 1.18 | Up |
| 21 | **Serine** | 78.317 (23.408) | 72.040 (50.547) | 0.0033 (W) | 0.0469 | 1.09 | Up |
| 22 | **PC aa C34:3** | 21.999 (9.086) | 18.097 (6.894) | 0.0039 (W) | 0.0487 | 1.22 | Up |
| 23 | **lysoPC a C20:3** | 1.316 (0.753) | 0.997 (0.649) | 0.0046 (W) | 0.0531 | 1.32 | Up |
| 24 | **lysoPC a C16:0** | 51.294 (18.532) | 42.485 (19.995) | 0.0062 (W) | 0.0618 | 1.21 | Up |
| 25 | **PC aa C36:6** | 0.727 (0.551) | 0.537 (0.298) | 0.0087 (W) | 0.0727 | 1.35 | Up |
| 26 | **PC aa C34:4** | 1.433 (0.934) | 1.100 (0.487) | 0.0130 (W) | 0.0931 | 1.3 | Up |
| 27 | **PC aa C40:5** | 10.124 (3.367) | 8.910 (3.182) | 0.0162 (W) | 0.106 | 1.14 | Up |
| 28 | **lysoPC a C16:1** | 1.769 (0.767) | 1.499 (0.790) | 0.0199 (W) | 0.115 | 1.18 | Up |
| 29 | **PC aa C36:1** | 48.497 (15.872) | 42.765 (13.531) | 0.0215 (W) | 0.1187 | 1.13 | Up |
| 30 | **PC aa C32:3** | 0.985 (0.384) | 0.849 (0.395) | 0.0222 (W) | 0.1187 | 1.16 | Up |
| 31 | **SM C18:0** | 27.504 (7.448) | 25.193 (11.277) | 0.0238 (W) | 0.1233 | 1.09 | Up |
| 32 | **PC aa C32:2** | 3.195 (1.907) | 2.581 (1.365) | 0.0275 (W) | 0.1377 | 1.24 | Up |
| 33 | **PC ae C38:3** | 7.169 (4.771) | 5.498 (3.085) | 0.0320 (W) | 0.153 | 1.3 | Up |
| 34 | **lysoPC a C17:0** | 0.949 (0.365) | 0.808 (0.348) | 0.0340 (W) | 0.153 | 1.18 | Up |
| 35 | **lysoPC a C18:2** | 12.795 (7.979) | 10.427 (7.151) | 0.0347 (W) | 0.153 | 1.23 | Up |
| 36 | **PC aa C36:2** | 224.131 (61.915) | 200.082 (57.420) | 0.0413 (W) | 0.1771 | 1.12 | Up |
| 37 | **Threonine** | 73.125 (28.811) | 66.382 (33.893) | 0.0481 (W) | 0.195 | 1.1 | Up |

**Table S7.** Unpaired t-test shows 37 known features significantly changed (p < 0.05, while 12 metabolites highlighted had a FDR< 0.05) between patients with severe pneumonia (PSI 4 and 5) vs. patients with less severe pneumonia (PSI 1, 2 and 3).


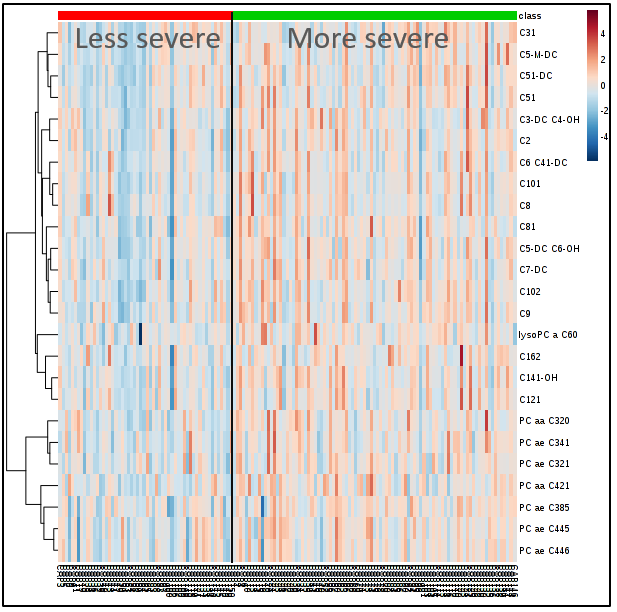


**Figure S11**. Heatmap analysis shows a separation between patients with more severe (PSI 4 and 5) and less severe (PSI 1, 2 and 3) CAP using the most differentiating metabolites by DI-MS/MS.

|  | **Name** | **Mean (SD) of ICU-admitted CAP** | **Mean (SD) of IUC ventilated controls** | **p-value** | **q-value (FDR)** | **Fold Change** | **CAP/ICU control** |
| --- | --- | --- | --- | --- | --- | --- | --- |
| 1 | **Mannose** | 0.069 (0.032) | 0.052 (0.016) | 0.0038 | 0.0104 | 1.33 | Up |
| 2 | **Valine** | 0.081 (0.034) | 0.068 (0.017) | 0.0295 | 0.0568 | 1.2 | Up |
| 3 | **3-Hydroxyisovalerate** | 0.002 (0.005) | 0.001 (0.000) | < 0.0001 (W) | 0.0001 | 3.43 | Up |
| 4 | **3-Methyl-2-oxovalerate** | 0.007 (0.004) | 0.004 (0.005) | < 0.0001 (W) | 0 | 1.92 | Up |
| 5 | **Acetoacetate** | 0.010 (0.009) | 0.044 (0.079) | < 0.0001 (W) | 0 | -4.6 | Down |
| 6 | **Adipate** | 0.004 (0.002) | 0.001 (0.000) | < 0.0001 (W) | 0 | 2.66 | Up |
| 7 | **Aspartate** | 0.019 (0.015) | 0.007 (0.004) | < 0.0001 (W) | 0 | 2.73 | Up |
| 8 | **Choline** | 0.006 (0.009) | 0.003 (0.002) | < 0.0001 (W) | 0.0001 | 1.92 | Up |
| 9 | **Dimethylamine** | 0.002 (0.003) | 0.001 (0.001) | < 0.0001 (W) | 0 | 3.09 | Up |
| 10 | **Fumarate** | 0.001 (0.001) | 0.001 (0.000) | < 0.0001 (W) | 0 | 2.68 | Up |
| 11 | **Hypoxanthine** | 0.004 (0.002) | 0.002 (0.001) | < 0.0001 (W) | 0 | 2.27 | Up |
| 12 | **Isopropanol** | 0.004 (0.007) | 0.018 (0.026) | < 0.0001 (W) | 0 | -4.35 | Down |
| 13 | **O-Phosphocholine** | 0.006 (0.010) | 0.001 (0.001) | < 0.0001 (W) | 0 | 4.16 | Up |
| 14 | **Phenylalanine** | 0.037 (0.028) | 0.020 (0.007) | < 0.0001 (W) | 0 | 1.81 | Up |
| 15 | **Proline** | 0.063 (0.051) | 0.032 (0.018) | < 0.0001 (W) | 0 | 1.96 | Up |
| 16 | **Urea** | 0.106 (0.068) | 0.046 (0.041) | < 0.0001 (W) | 0 | 2.29 | Up |
| 17 | **Tyrosine** | 0.031 (0.015) | 0.021 (0.006) | 0.0001 (W) | 0.0004 | 1.46 | Up |
| 18 | **Beta-Alanine** | 0.006 (0.006) | 0.003 (0.002) | 0.0002 (W) | 0.0005 | 1.84 | Up |
| 19 | **Isobutyrate** | 0.006 (0.006) | 0.005 (0.007) | 0.0008 (W) | 0.0023 | 1.37 | Up |
| 20 | **Succinate** | 0.009 (0.028) | 0.003 (0.002) | 0.0009 (W) | 0.0025 | 3.44 | Up |
| 21 | **Ornithine** | 0.018 (0.011) | 0.011 (0.006) | 0.0055 (W) | 0.0143 | 1.61 | Up |
| 22 | **Methionine** | 0.014 (0.011) | 0.009 (0.004) | 0.0061 (W) | 0.0151 | 1.61 | Up |
| 23 | **Arginine** | 0.044 (0.022) | 0.032 (0.012) | 0.0078 (W) | 0.0185 | 1.38 | Up |
| 24 | **Betaine** | 0.026 (0.019) | 0.017 (0.007) | 0.0089 (W) | 0.0201 | 1.5 | Up |
| 25 | **Creatinine** | 0.047 (0.046) | 0.030 (0.025) | 0.0103 (W) | 0.0223 | 1.58 | Up |
| 26 | **4-Hydroxybutyrate** | 0.011 (0.012) | 0.010 (0.015) | 0.0174 (W) | 0.0362 | 1.15 | Up |
| 27 | **2-Oxoisocaproate** | 0.004 (0.002) | 0.003 (0.002) | 0.0193 (W) | 0.0386 | 1.38 | Up |
| 28 | **2-Hydroxyisovalerate** | 0.004 (0.003) | 0.003 (0.002) | 0.0347 (W) | 0.0644 | 1.48 | Up |
| 29 | **Creatine** | 0.033 (0.052) | 0.036 (0.055) | 0.0372 (W) | 0.0666 | -1.11 | Down |
| 30 | **Histidine** | 0.025 (0.011) | 0.020 (0.008) | 0.0408 (W) | 0.0708 | 1.27 | Up |

**Table S8.** Unpaired t-test shows 27 metabolites obtained by NMR significantly changed (highlighted FDR< 0.05) between ICU-admitted bacterial CAP and ICU ventilated controls.

|  | **Metabolites** | **Mean (SD) of CAP** | **Mean (SD) of ICU** | **p-value** | **q-value (FDR)** | **Fold Change** | **CAP/ICU** |
| --- | --- | --- | --- | --- | --- | --- | --- |
| 1 | **Phosphoric acid 3T** | 2689.548 (3065.588) | 221.935 (183.181) | < 0.0001 (W) | 0 | 12.12 | Up |
| 2 | **Urea 2TMS** | 1878074.984 (1370702.283) | 8673.857 (6881.056) | < 0.0001 (W) | 0 | 216.52 | Up |
| 3 | **Lactic acid 2TMS** | 17982.965 (15227.908) | 2228.968 (618.786) | < 0.0001 (W) | 0 | 8.07 | Up |
| 4 | **Iminodiacetic acid 3TMS** | 3939.387 (5016.916) | 1005.468 (366.005) | < 0.0001 (W) | 0 | 3.92 | Up |
| 5 | **Butanoic acid, 2-hydroxy- 2TMS** | 31104.847 (38829.780) | 4561.097 (1882.466) | < 0.0001 (W) | 0 | 6.82 | Up |
| 6 | **Mannitol 6TMS** | 19933.661 (40396.609) | 1848.853 (1492.624) | < 0.0001 (W) | 0 | 10.78 | Up |
| 7 | **Altrose 1MEOX 5TMS BP** | 386333.746 (583776.143) | 8989.645 (7665.971) | < 0.0001 (W) | 0 | 42.98 | Up |
| 8 | **NA Inositol like** | 23516.739 (35087.081) | 1962.242 (587.282) | < 0.0001 (W) | 0 | 11.98 | Up |
| 9 | **Fructose 1MEOX 5TMS BP** | 17533.837 (25079.903) | 2023.258 (1616.822) | < 0.0001 (W) | 0 | 8.67 | Up |
| 10 | **Arabinose 1MEOX 4TMS MP** | 1827.254 (1481.786) | 561.935 (408.760) | < 0.0001 (W) | 0 | 3.25 | Up |
| 11 | **Tricosane, n-** | 1498.517 (2222.095) | 2776.757 (2375.275) | < 0.0001 (W) | 0 | -1.85 | Down |
| 12 | **Eicosanoic acid 1TMS** | 5241.370 (4823.033) | 2291.073 (764.625) | < 0.0001 (W) | 0.0001 | 2.29 | Up |
| 13 | **Octadecadienoic acid, 9,12-Z,Z- 1TMS** | 1355.915 (1388.412) | 6331.748 (2033.010) | < 0.0001 (W) | 0 | -4.67 | Down |
| 14 | **Octadecenoic acid, 9-E- 1TMS** | 1127.528 (967.131) | 2281.364 (1367.618) | < 0.0001 (W) | 0 | -2.02 | Down |
| 15 | **Octadecanoic acid 1TMS** | 471192.937 (236468.039) | 274319.416 (45998.773) | < 0.0001 (W) | 0 | 1.72 | Up |
| 16 | **Aminomalonic acid 3TMS** | 2735.647 (2704.425) | 293.847 (330.707) | < 0.0001 (W) | 0 | 9.31 | Up |
| 17 | **Pyridine, 2-hydroxy- 1TMS** | 5619.717 (5392.262) | 636.804 (846.419) | < 0.0001 (W) | 0.0002 | 8.82 | Up |
| 18 | **Benzoic acid, 1TMS** | 121497.927 (175205.961) | 2474.123 (1669.032) | < 0.0001 (W) | 0 | 49.11 | Up |
| 19 | **Glucuronic acid 1MEOX 5TMS MP** | 8308.664 (11699.362) | 113.952 (90.663) | < 0.0001 (W) | 0 | 72.91 | Up |
| 20 | **Ribitol 5TMS** | 2157.926 (2104.971) | 561.935 (408.760) | < 0.0001 (W) | 0 | 3.84 | Up |
| 21 | **Lactic acid 2TMS** | 464060.335 (367622.354) | 10454.893 (9627.411) | < 0.0001 (W) | 0 | 44.39 | Up |
| 22 | **Threonic acid 4TMS** | 6183.238 (6765.939) | 1964.823 (897.002) | < 0.0001 (W) | 0 | 3.15 | Up |
| 23 | **Phosphoric acid 3TMS** | 137720.091 (130724.772) | 425559.234 (222656.234) | < 0.0001 (W) | 0 | -3.09 | Down |
| 24 | **Glyceric acid 3TMS** | 4178.349 (2836.060) | 525.306 (228.039) | < 0.0001 (W) | 0 | 7.95 | Up |
| 25 | **Pyruvic acid 1MEOX 1TMS** | 10656.839 (12776.971) | 2321.081 (1019.024) | < 0.0001 (W) | 0.0001 | 4.59 | Up |
| 26 | **Glycerol 3TMS** | 74693.623 (54822.937) | 12973.656 (5696.659) | < 0.0001 (W) | 0 | 5.76 | Up |
| 27 | **Galactose 1MEOX 5TMS BP** | 805962.914 (392022.382) | 2338.638 (1618.339) | < 0.0001 (W) | 0 | 344.63 | Up |
| 28 | **Galactose 1MEOX 5TMS MP** | 117915.313 (84637.924) | 11727.365 (9750.297) | < 0.0001 (W) | 0 | 10.05 | Up |
| 29 | **Glucose 1MEOX 5TMS MP** | 852064.666 (930927.454) | 12596.562 (10017.093) | < 0.0001 (W) | 0 | 67.64 | Up |
| 30 | **Glutamic acid 3TMS** | 37649.402 (46378.112) | 169.484 (143.709) | < 0.0001 (W) | 0 | 222.14 | Up |
| 31 | **Glycine 3TMS** | 30314.806 (22469.554) | 1412.746 (2768.616) | < 0.0001 (W) | 0 | 21.46 | Up |
| 32 | **Alanine 2TMS** | 152712.294 (152414.609) | 509.383 (560.367) | < 0.0001 (W) | 0 | 299.8 | Up |
| 33 | **Homoserine 4TMS** | 2319.682 (1876.499) | 802.824 (774.100) | < 0.0001 (W) | 0.0001 | 2.89 | Up |
| 34 | **Threonine 3TMS** | 7140.942 (5144.938) | 352.952 (272.326) | < 0.0001 (W) | 0 | 20.23 | Up |
| 35 | **Tryptophan 3TMS** | 3518.448 (3303.573) | 868.000 (547.060) | < 0.0001 (W) | 0 | 4.05 | Up |
| 36 | **Phenylalanine 2TMS** | 65121.149 (42848.774) | 893.597 (756.133) | < 0.0001 (W) | 0 | 72.88 | Up |
| 37 | **Hexadecanoic acid 1TMS** | 244612.472 (117983.950) | 161764.652 (28531.649) | 0.0005 (W) | 0.0009 | 1.51 | Up |
| 39 | **Mannose 1MEOX 5TMS BP** | 444527.340 (698809.298) | 11675.548 (9712.119) | 0.0010 (W) | 0.0016 | 38.07 | Up |
| 40 | **Sucrose 8TMS** | 15759.030 (37100.610) | 1966.739 (3900.308) | 0.0020 (W) | 0.0032 | 8.01 | Up |
| 41 | **Ornithine 3TMS** | 1221.104 (1209.919) | 680.952 (544.444) | 0.0057 (W) | 0.009 | 1.79 | Up |
| 42 | **Butanoic acid, 3-hydroxy- 2TMS** | 28925.651 (41416.573) | 5940.645 (1721.436) | 0.0082 (W) | 0.0128 | 4.87 | Up |
| 43 | **Alanine 2TMS** | 1399.023 (1082.856) | 759.629 (349.702) | 0.0107 (W) | 0.0162 | 1.84 | Up |
| 44 | **Alanine, beta- 1TMS** | 13716.221 (15655.692) | 3808.274 (404.882) | 0.0129 (W) | 0.0189 | 3.6 | Up |
| 45 | **Octanoic acid, n- 1TMS** | 2464.424 (2041.411) | 1516.468 (694.814) | 0.0173 (W) | 0.0247 | 1.63 | Up |
| 46 | **Octadecenoic acid, 9-Z- 1TMS** | 13303.736 (33346.203) | 5619.363 (2320.946) | 0.0454 (W) | 0.0609 | 2.37 | Up |

**Table S9.** Unpaired t-test shows 46 known features obtained by GC-MS significantly changed (FDR< 0.05) between ICU-admitted bacterial CAP and ICU ventilated controls.

| Analytical  platform | R^2^Y | Q^2^Y | P value | Sensitivity | Specificity | AUROC |
| --- | --- | --- | --- | --- | --- | --- |
| DI-MS/MS | 0.445 | 0.299 | 1.1x10^-8^ | 82 | 91 | 0.91 |
| GC-MS | 0.36 | 0.189 | 0.01 | 75 | 82 | 0.79 |
| NMR | 0.31 | 0.156 | 0.008 | 72 | 75 | 0.71 |

**Table S10**. Comparison of three analytical platforms shows that DI-MS/MS is more predictive and significant for 90 day mortality than GC-MS and NMR.

| **Variables** | **ICU admitted (n=40)** | **Non-ICU admitted (n=40)** |
| --- | --- | --- |
| Age yrs. (mean ± SD) | 76.1 ± 7.5 | 76.1 ± 9.2 |
| Male/Female | 20/20 | 20/20 |
| Weight (mean ± SD) | 161.1 ± 34.4 | 159.1 ± 39.4 |
| Hospital LOS | 12.7 ± 8.9 | 7.2 ± 3.1 * |
| ICU LOS | 5.03 ± 4.08 | 0 * |
| APACHE III | 74.2 ± 3.2 | 64.3 ± 3.2 * |
| PSI (Day 0) | 100.4 ± 46.1 | 90.7 ± 49.5 |
| PSI (Day 1) | 135 ± 38.1 | 119.4 ± 40.5 |
| PSI (Day 1 NoAge) | 63.9 ± 36.4 | 47.8 ± 38.5 |
| Mechanical ventilation ^a^ | 4 (10) | 0 |
| Noninvasive ventilation ^a^ | 5 (12.5) | 0 |
| Comorbidities ^a^  Other respiratory diseases  Neoplastic diseases  Neurological diseases  Aids  Sepsis  Liver disease  CHF  Cerebrovascular disease  Renal disease  Altered mental status | 16 (40)  2 (5)  3 (7.5)  0 (0)  13 (32.5)  1 (2.5)  9 (22.5)  4 (10)  3 (7.5)  6 (15) | 13 (32)  4 (10)  17 (22)  0 (0)  11 (27.5)  0 (0)  8 (24)  2 (5)  1 (2.5)  3 (7.5) |
| Smoker | 23 (57) | 29 (72) |
| Alcoholism | 7 (17.5) | 5 (12.5) |
| Clinical manifestation ^a^  Lowest temperature (°C)  Highest temperature (°C)  Pulse ≥ 125/min  BUN ≥ 30 mg/dl  Respiratory >30 rate/min  PaO_2_ < 60 mm/Hg  pH < 7.35  Lowest systolic BP (mm/Hg)  Highest systolic BP (mm/Hg)  Highest creatinine (mg/dL) | 36.21 ± 0.74  37.80 ± 0.8  8 (20)  11 (27.5)  8 (20)  15 (37.5)  3 (7.5)  117 ± 16  144 ± 26  1.2 ± 0.71 | 36.5 ± 1.6  37.45 ± 0.81  3 (7.5)  11 (27.5)  4 (10)  10 (25)  2 (5)  117 ± 21  147 ± 22  1.6 ± 1.42 |

**Table S11.** The characteristics of CAP patients who were admitted to ICU vs. CAP patients who were not admitted to ICU.
